# Evolutionary and Functional Analysis of Old World Primate TRIM5 Reveals the Ancient Emergence of Primate Lentiviruses and Convergent Evolution Targeting a Conserved Capsid Interface

**DOI:** 10.1371/journal.ppat.1005085

**Published:** 2015-08-20

**Authors:** Kevin R. McCarthy, Andrea Kirmaier, Patrick Autissier, Welkin E. Johnson

**Affiliations:** 1 Harvard Program in Virology, Harvard Medical School, Boston, Massachusetts, United States of America; 2 Biology Department, Boston College, Chestnut Hill, Massachusetts, United States of America; Fred Hutchinson Cancer Research Center, UNITED STATES

## Abstract

The widespread distribution of lentiviruses among African primates, and the lack of severe pathogenesis in many of these natural reservoirs, are taken as evidence for long-term co-evolution between the simian immunodeficiency viruses (SIVs) and their primate hosts. Evidence for positive selection acting on antiviral restriction factors is consistent with virus-host interactions spanning millions of years of primate evolution. However, many restriction mechanisms are not virus-specific, and selection cannot be unambiguously attributed to any one type of virus. We hypothesized that the restriction factor TRIM5, because of its unique specificity for retrovirus capsids, should accumulate adaptive changes in a virus-specific fashion, and therefore, that phylogenetic reconstruction of TRIM5 evolution in African primates should reveal selection by lentiviruses closely related to modern SIVs. We analyzed complete TRIM5 coding sequences of 22 Old World primates and identified a tightly-spaced cluster of branch-specific adaptions appearing in the Cercopithecinae lineage after divergence from the Colobinae around 16 million years ago. Functional assays of both extant TRIM5 orthologs and reconstructed ancestral TRIM5 proteins revealed that this cluster of adaptations in TRIM5 specifically resulted in the ability to restrict Cercopithecine lentiviruses, but had no effect (positive or negative) on restriction of other retroviruses, including lentiviruses of non-Cercopithecine primates. The correlation between lineage-specific adaptations and ability to restrict viruses endemic to the same hosts supports the hypothesis that lentiviruses closely related to modern SIVs were present in Africa and infecting the ancestors of Cercopithecine primates as far back as 16 million years ago, and provides insight into the evolution of TRIM5 specificity.

## Author Summary

Old World primates in Africa are reservoir hosts for more than 40 species of simian immunodeficiency viruses (SIVs), including the sources of the human immunodeficiency viruses, HIV-1 and HIV-2. To investigate the prehistoric origins of these lentiviruses, we looked for patterns of evolution in the antiviral host gene *TRIM5* that would reflect selection by lentiviruses during evolution of African primates. We identified a pattern of adaptive changes unique to the TRIM5 proteins of a subset of African monkeys that suggests that the ancestors of these viruses emerged between 11–16 million years ago, and by reconstructing and comparing the function of ancestral TRIM5 proteins with extant TRIM5 proteins, we confirmed that these adaptations confer specificity for their modern descendants, the SIVs.

## Introduction

The lentiviruses comprise a genus within the family Retroviridae [1]. These include viruses of horses, small ruminants, cows and felids, as well as some 40 or more species of primate lentiviruses- the latter including HIV-1, HIV-2 and the simian immunodeficiency viruses (SIVs) of Old World African primates [2]. The primate lentiviruses form a distinct branch within the *lentivirus* genus, and share a number of derived features including several unique accessory genes [3]. Endogenous sequences related to the modern lentiviruses have been discovered in the genomes of mustelids (weasels and ferrets) [4, 5], lagomorphs (rabbits and hares) [6, 7], colugos (flying lemurs) [8] and multiple species of lemur [9, 10]. These ancient lentivirus ERVs (endogenous retroviruses) interleave with modern lentiviruses in phylogenetic trees, and molecular clock analyses indicate that they range in age from 3 to 12 million years [3]. One of these, the pSIVgml ERV of lemurs, shares features with both non-primate and primate lentiviruses, and therefore represents a transitional form bridging the primate and non-primate lentiviruses [3, 9]. These observations indicate that lentiviruses very similar to modern lentiviruses have existed for at least several million years. However, it remains an open question as to when the common ancestors of the modern primate lentiviruses first emerged in the ancestors of extant African primates, and whether emergence of these viruses influenced evolution of host antiviral defense genes.

There are several observations suggesting that lentiviruses have been endemic to African primates going back many generations. For example, there is a general trend in which pathogenic infections are associated with a recent acquisition of a primate lentivirus. Thus, higher mortality rates are observed among humans infected with HIV-1 or HIV-2, chimpanzees infected with SIVcpz, rhesus macaque species experimentally infected with SIVmac or SIVsmm, and pig-tailed macaques infected with SIVagm [11–18]. In contrast, primates believed to be long-standing hosts of a particular SIV are less likely to present overt clinical symptoms of infection during a normal lifespan [12, 13, 19]. However, the timescales required for coevolution to result in non-pathogenic interactions between lentiviruses and hosts are unknown; therefore, while such observations are suggestive of long-term coevolution, they are not useful for dating the origins of these viruses. Comparisons of extant SIVs on mainland Africa and Bioko Island suggest that modern SIVs were present for at least the past 30,000 years [20]. In contrast, endogenous lentivirus sequences establish that lentiviruses existed 3–15 million years ago, and the identification and functional analysis of positively selected sites in certain host-encoded restriction factors also provides compelling, indirect evidence for the existence of ancient lentiviruses [21–28].

TRIM5 is unique among well-characterized restriction factors in specifically targeting retroviruses via direct binding to the viral capsid after entry into the cytoplasm of the infected cell [29–31]. As a consequence, evolutionarily derived changes in *TRIM5* of modern species should include adaptations selected by retroviruses encountered by the ancestors of those same species [32]. We therefore reasoned that reconstructing the evolution of the *TRIM5* gene of African Catarrhine primates (Old World monkeys and apes) should reveal patterns consistent with long-term interactions with primate lentiviruses, and that host lineage-specific adaptations in TRIM5 should correlate specifically with recognition and restriction of extant primate lentiviruses. Here, we used additional sampling of TRIM5 sequences from Old World primates, phylogenetic reconstruction, and restriction of a panel of retroviruses representing multiple retroviral genera to establish a correlation between 1) adaptations unique to the *TRIM5* gene of Cercopithecine monkeys (macaques, mangabeys, baboons, guenons, African green monkeys, and other related species) but not other primates, and 2) specificity for only that subset of primate lentiviruses endemic to modern Cercopithecinae hosts. The distribution of these changes on a phylogeny of African primates indicates that ancestral lentiviruses closely related to modern SIVs began colonizing the primate lineage in Africa as far back as 11–16 million years ago. Furthermore, using a panel of previously described amino-acid substitutions in the SIVmac239 capsid protein (CA), we found that the TRIM5 proteins of two different Cercopithecinae lineages evolved to target an interface unique to the capsid proteins of lentiviruses.

## Results

### Unique lineage-specific adaptations in the V1 domain of TRIM5α in Cercopithecinae monkeys

TRIM proteins are named for their shared tripartite domain structure comprising RING, B-box and coiled-coil domains [33, 34]. The α isoform of TRIM5 encodes a C-terminal PRYSPRY domain that acts as the viral recognition domain [29, 30]. Among primates, the TRIM5α PRYSPRY domain has evolved under strong positive selection with a majority of positively selected sites clustered within four variable domains (V1 to V4) [25, 35, 36]. These variable domains are thought to directly mediate contacts with retroviral capsids (CA) [35–52]. Notably, the length of the V1 sequence has remained constant in all primate lineages except the Cercopithecinae [29, 36, 53, 54]. The subfamily Cercopithecinae includes two tribes, the Cercopithicini (including guenons, Patas monkeys and African green monkeys) and the Papionini (which includes macaques, baboons, and mangabeys); V1 length variation in the Cercopithecines differs between the two tribes, with some Papionini TRIM5α V1s having been lengthened by two amino acids, while some Cercopithicini TRIM5α V1s have been lengthened by 20 amino acids [29, 36, 53].

**Fig 1:**
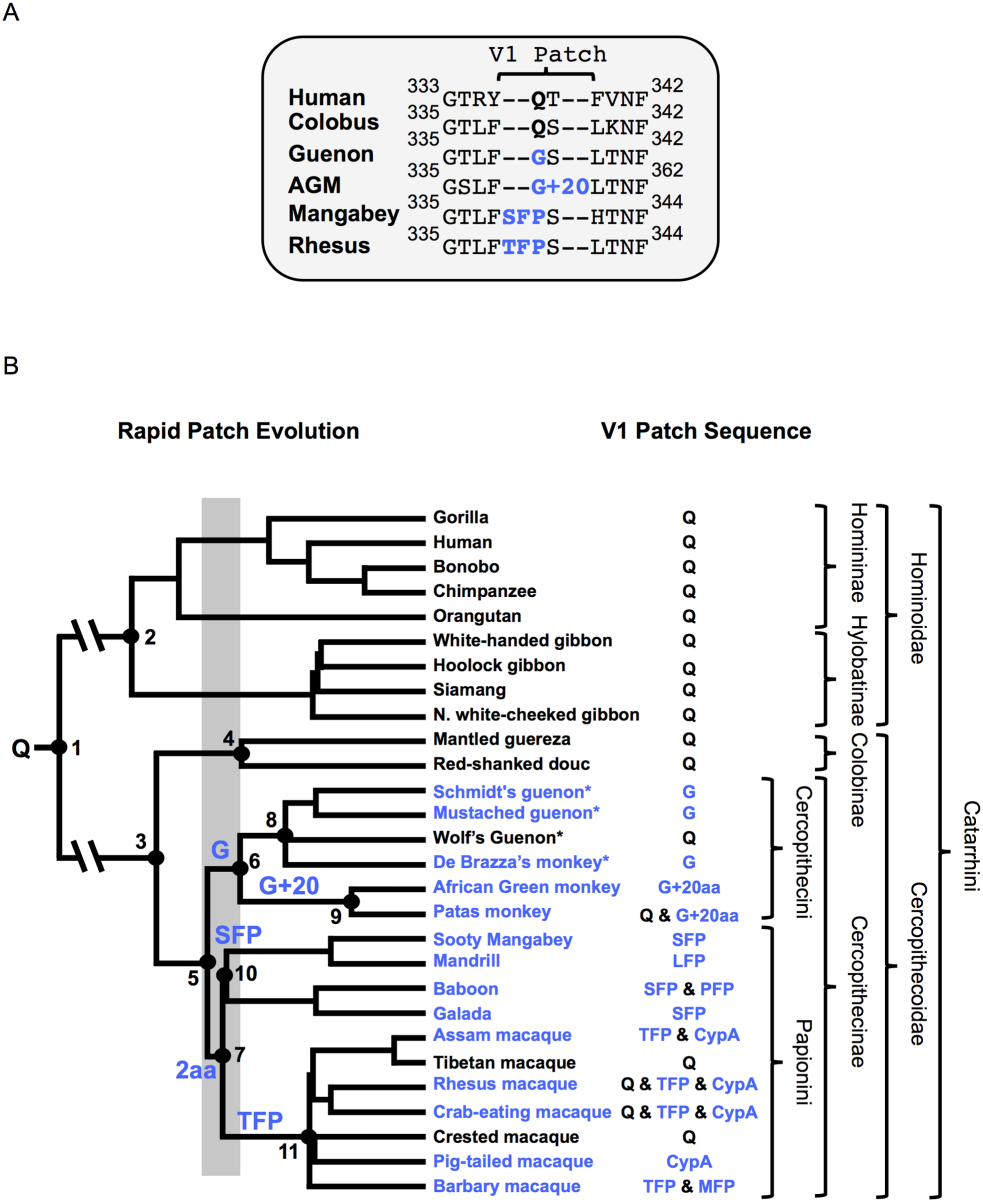
Phylogeny and TRIM5α V1-patch sequences of Old World primates. (A) Sequence alignment of select Catarrhini TRIM5α variable loop 1 sequences. The rapidly evolving V1-patch is indicated and modifications replacing V1:Q (bold) are highlighted in blue. (B) Phylogeny of select Old World primates and key evolutionary events in TRIM5α evolution. Nodes of interest are numbered and corresponding date ranges for each node are provided in Table 1. Inferred events in the evolution of the V1-patch are written above the node/branch on which they occurred. A gray box corresponds to the time period in which V1:Q was replaced by a G (Cercopithecini) or by a 2 amino-acid insertion (Papionini). Blue lettering corresponds to modifications that replaced V1:Q. Common species names are indicated at the branch tips. An “*” indicates full-length TRIM5α sequences first reported in this manuscript. The feature found in the V1-patch of each species is provided. Relevant taxonomic classifications are indicated. The tree is adapted from Bininda-Emonds et al., 2007 [55].

To reconstruct the evolution of V1 sequences in Cercopithecinae primates, we generated an alignment that included TRIM5 sequences retrieved from public databases and by sequencing of previously unreported *TRIM5* genes representing five additional Cercopithecinae species, for a total of 22 Catarrhini species and subspecies. These included new sequences representing four guenon species, *Cercopithecus wolfi* (Wolf’s Guenon, n = 3), *Cercopithecus cephus* (mustached guenon, n = 1), *Cercopithecus ascanius* (Schmidt’s guenon n = 2), *Cercopithecus neglectus* (De Brazza's monkey, n = 1), and the mangabey *Cercocebus torquatus* (red-capped mangabe*y*, n = 1). We then used this alignment to trace the origins of the V1 length variants and map evolutionary events onto the established phylogeny of Old World primates (Fig 1).

Consistent with previous reports, we found that length variation in the TRIM5 V1 region is unique to the Cercopithecinae. Among some cercopithecine primates, at least two independent duplication-insertion events occurred at or adjacent to TRIM5α amino acid position 339. Due to the length polymorphisms in this region we will refer to this site, centered on cercopithecine TRIM5α position 339, as the V1-patch (Fig 1). From our analysis we established that a Q (henceforth “V1:Q”) corresponding to position 339 in cercopithecine TRIM5αs represents the state that was present in the ancestor of all Old World primates ≥30 million years ago (Fig 1 and Table 1). Strikingly, over the course of catarrhine evolution, V1:Q has remained unmodified in all other primate lineages except the Cercopithecinae. In contrast, among the latter we found TRIM5 variants with the evolutionarily derived V1 modifications at this site, in addition to TRIM5 orthologs that had retained the ancestral V1:Q residue (Fig 1B). The presence of the ancestral (V1:Q) and derived residues in the Cercopithecinae indicates that selection favored the maintenance of multiple TRIM5α variants in this primate lineage, a possible indication of long-term balancing selection [54].

**Table 1:** Estimated dates for nodes of interest. A table of estimated dates for nodes of interest in Fig 1. Reported estimated dates of last common ancestors (LCA) provided in millions of years ago (MYA) are from the following studies: Bininda-Emonds et al. (2007) [55], Perelman et al. (2011) [56], Pozzi et al. (2014) [57], Fabre et al. (2009), Finstermeier et al. (2013) [58]. An “-”denotes a date that was not estimated.

Event	Node	Bininda-Emonds et al. (2007) In MYA	Perelman et al. (2011) In MYA	Pozzi et al. (2014) In MYA	Fabre et al. (2009) In MYA	Finstermeier et al. (2013) In MYA
LCA Catarrhini	1	34.4	31.56	32.12	23.9	31.91
LCA Hominoidea	2	21.4	20.32	22.32	18.6	20.31
LCA Cercopithecoidea	3	19.7	17.57	20.82	-	22.83
LCA Colobinae	4	13.5	12.28	14.11	8.7	11.86
LCA Cercopithecinae	5	15.9	11.5	14.09	13.3	14.85
LCA Cercopithecini	6	13.8	8.22	11.19	9.7	9.53
LCA Papionini	7	14.8	8.13	12.17	7.4	11.35
LCA Guenons	8	10.5	7.28	9.03	-	-
LCA Patas/Green Monkeys	9	5.5	7.63	3.56	-	8.91
LCA Baboon/mangabeys	10	14.6	6.67	-	-	10.76
LCA macaques	11	8.5	5.12	4.42	-	7.4

All V1-patch variants representing the Cercopithecini tribe either have a Q at position 339 (or the homologous position), or else they share a common V1:Q-to-G substitution (which we call V1:G). In addition, the cercopithecin V1:G variant was further modified by a duplication of adjacent sequence resulting in the insertion of 20 additional amino acids (which we call V1:G+20); examples of extant species with the V1:G+20 variant include African green monkeys and some Patas monkeys (Fig 1) [36, 53]. Interspecies and intraspecies differences within these 20 residues indicate that the inserted sequences have continued to evolve after the initial duplication event. The presence of a G at the homologous position in TRIM5α of guenons, African green monkeys and some Patas monkeys allowed us to date both evolutionary events (Fig 1A). Thus, we infer that V1:G arose first, between ~11 and 16 million years ago, while the insertion event leading to V1:G+20 likely occurred between ~3.5 and 14 million years ago (Fig 1B and Table 1).

In the Papionini, all variants of V1:Q share a two-amino-acid duplication (Fig 1B); among the Papionini, there are examples of TRIM5αs with this two-amino-acid insertion in every genus. This indicates that the original insertion was present in the last common ancestor of all extant papionin species ~8–15 million years ago (Fig 1B). Following the insertion event, this modified patch continued to evolve, resulting in the V1:SFP, V1:TFP, V1:PFP, V1:LFP, and V1:MFP derivatives found in extant papionin species (we will use “V1:+2” when referring to these collectively) (S1A Fig). These changes to the two-amino-acid duplication have obscured the sequence of the initial insertion and its potential evolutionary intermediates. From an examination of known papionin TRIM5α sequences and the papionin phylogeny, we have inferred one possible evolutionary pathway (illustrated in S1 Fig). Briefly, the insertion most likely arose from a six-base pair duplication of adjacent sequence resulting in V1:QFQ, which subsequently underwent a number of substitutions (S1B and S1C Fig).

We and others have previously reported that Asian macaques have a third TRIM5 variant, in which the PRYSPRY domain of a V1:Q ortholog has been replaced by a cyclophilin A domain (Fig 1B); thus far, the TRIM5-cyclophilin A fusion has not been shown to restrict any viruses other than lentiviruses [59–63].

Diversity within the V1-patch is unusual among other Old World primates, making the appearance of multiple V1-patch modifications in the Cercopithecinae remarkable. In contrast to V1:Q, which has remained unmodified outside the Cercopithecinae for more than 30 million years, within the Cercopithecinae the V1-patch was modified by evolution at least twice, in two independent lineages (the Cercopithecini and the Papionini tribes) in the space of about 1–4 million years, about 8–16 million years ago (Fig 1). These V1 modifications then continued to evolve, with further modifications occurring at or adjacent to position 339 in V1 (Fig 1).

### Adaptations in the TRIM5 variable loop 1 patch of cercopithecine species specifically affect restriction of modern cercopithecine SIVs, but not other retroviruses

The emergence of V1 patch variants in two independent Cercopithecinae lineages suggests that these early modifications may have conferred a selective advantage. We therefore next sought to determine the impact, if any, that these modifications have on the restriction of retroviruses. To do this, we assayed for restriction by a panel of TRIM5 orthologs, including a recreated ancestral TRIM5α sequence representing the last common ancestor of all cercopithecine TRIM5 sequences.

First, in order to reconstruct the sequence of an ~11–16 million year old TRIM5α protein to represent the last common ancestor of all extant TRIM5s of the subfamily Cercopithecinae, we created a comprehensive sequence alignment that included 60 full-length TRIM5α sequences from 22 primate species and subspecies. A maximum-likelihood tree was generated from this alignment (S2 Fig). Both the alignment and tree were used to predict the sequence of the last common ancestral sequence of all cercopithecine TRIM5αs using the FastML server (http://fastml.tau.ac.il/) [64–66]. This reconstructed TRIM5α, which we refer to as ancTRIM5α^V1:Q^, is predicted to reflect the sequence that predates the selective events at TRIM5α V1 position 339 (includes the ancestral Q at position 339) (S2 and S3 Figs). AncTRIM5α^V1:Q^ approximates the ancestral TRIM5α sequence in which the initial V1 adaptations occurred, and importantly, provides a single, isogenic backbone for directly comparing the functional consequences of individual V1 adaptations in the context of an otherwise identical protein sequence. In combination with the panel of naturally occurring TRIM5 orthologs bearing these adaptations, this allows us to make a thorough assessment of the impact of these changes on restriction of a diverse panel of retroviruses.

**Fig 2:**
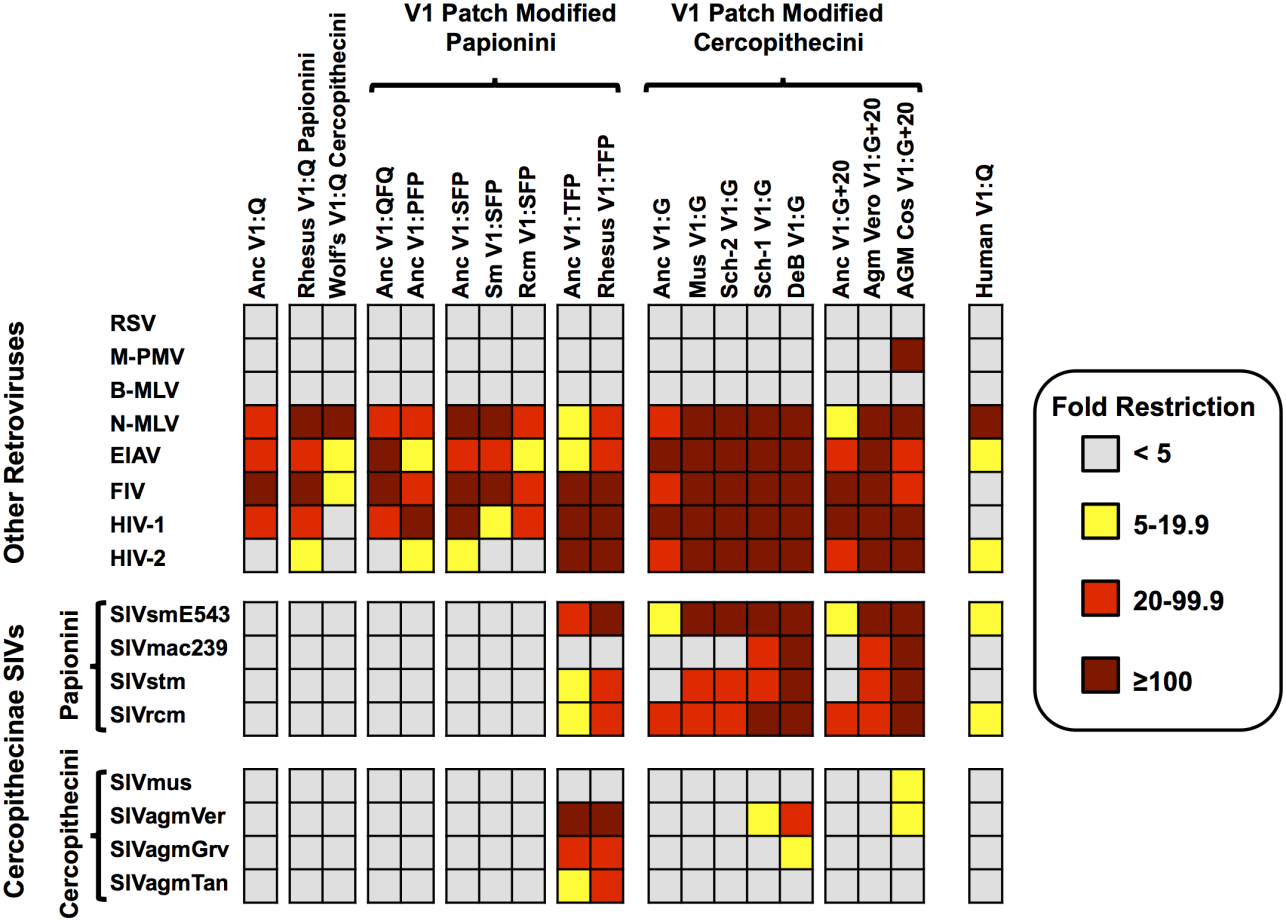
Patterns of restriction by ancient TRIM5αs mirror those of modern TRIM5αs with matched V1 features. Restriction by the ancestral, modified-ancestral, and modern TRIM5αs as measured against a diverse panel of non-lentiviruses and lentiviruses. Color corresponds to fold restriction as indicated by the key. The features found in the V1-patch are indicated. Each data point is the average of at least three independent experiments. Specific values for each box can be found in S1 Dataset.

Thus, we also modified ancTRIM5α^V1:Q^ to compare the impact of each of the cercopithecine V1 adaptations, including the V1:G and V1:G+20 variants, which we refer to as ancTRIM5α^V1:G^ and ancTRIM5α^V1:G+20^. The 20 amino-acid insertion recreates the original duplication (that is, the duplicated sequences are identical) without the additional diversification seen in extant TRIM5α orthologs. We also generated ancTRIM5α derivatives with the additional V1-patch modifications found in Papionini species (ancTRIM5α^V1:SFP^ and ancTRIM5α^V1:TFP^) and their predicted evolutionary intermediates (ancTRIM5α^V1:QFQ^, ancTRIM5α^V1:PFP^)(Fig 2 and S1 Fig).

We next generated stable cell lines expressing each of the HA-tagged ancTRIM5α^V1:Q^ derivatives, as well as cell lines stably expressing HA-tagged versions of TRIM5α orthologs cloned from extant cercopithecine species. The latter included TRIM5α orthologs with a naturally occurring G at position 339 from mustached guenons (mus), De Brazza’s monkeys (deb) and two alleles from Schmidt’s guenon (Sch1 and Sch2), and TRIM5α orthologs bearing the 339G and the 20-amino-acid insertion from African green monkey-derived Vero cells (AgmV) and COS-1 cells (AgmC). TRIM5αs with the V1:SFP modification came from sooty mangabeys and red-capped mangabeys (sm (ceat-1) and rcm, respectively). Rhesus (rh) TRIM5α (mamu-1) is a modern day V1:TFP allele. Cercopithecine TRIM5α orthologs with an unmodified V1:Q were included from rhesus macaque (mamu-5) and Wolf’s guenon (wlf) TRIM5αs. Human (hu) TRIM5α (V1:Q) was included as a non-cercopithecine control (Fig 2).

To ask whether adaptations in TRIM5 V1 unique to cercopithecine primates may have been selected specifically for recognition of Cercopithecine retroviruses, we sought to determine whether the V1:G, V1:+2 and V1:G+20 variants and their derivatives specifically affect restriction of cercopithecine SIVs, or whether they have more general effects, either positive or negative, on the ability to restrict other retroviruses. Specifically, to assay restriction by our panel of 19 TRIM5α proteins, we assembled a representative panel of cercopithecine SIVs, as well as two human lentiviruses (HIV-1nl4.3 and HIV-2rod), two non-primate lentiviruses (the feline immunodeficiency virus, FIV, and the equine infectious anemia virus, EIAV); an avian alpharetrovirus (RSV); two murine gammaretroviruses (N-tropic and B-tropic strains of murine leukemia virus, N-MLV and B-MLV); and a betaretrovirus (Mason-Pfizer monkey virus, M-PMV).

Infectious SIV virus stocks were produced either by transfection of the respective molecular clones or of HIV-1-based molecular clones engineered to encode the CA domains of other Cercopithecine SIVs (S4–S6 Figs). These included the SIVs from species in the tribe Cercopithicini—mustached guenons (SIVmus) and African green monkeys (SIVagmTan-1, SIVagmVer, and SIVagmGrv)—and from the Papionini tribe- macaque-passaged sooty mangabey SIV (SIVsmE543-3), the rhesus macaque SIV isolate (SIVmac239), a stump tailed macaque SIV (SIVstm) and an SIV isolate from a red-capped mangabey (SIVrcm) (Fig 2).

The 19 TRIM5-expressing cell lines were assayed for the ability to restrict each of the 16 viruses in the panel. Although expression levels of the different TRIM5α derivatives varied, we did not observe a correlation between expression level and restriction (Fig 2 and S7 Fig, S1 Dataset). Importantly, all TRIM5α constructs, including the synthetic ancestral constructs, expressed functional TRIM5α proteins, as each of the 19 cell lines was able to restrict three or more viruses representing two or more retroviral genera (Fig 2).

Other than the avian alpharetrovirus (RSV) and the mouse gammaretrovirus B-MLV, which were not restricted by any of the 19 TRIM5α proteins tested, all other viruses were restricted by at least one TRIM5-expressing cell line. Except for the Cercopithecine SIVs, all of the other retroviruses were either almost always resistant (RSV, M-PMV, B-MLV) or almost always sensitive (N-MLV, EIAV, FIV and HIV-1) to restriction by the majority of the TRIM5α proteins tested (Fig 2). For example, N-MLV was restricted by all 19 TRIM5α proteins in the panel, whereas B-MLV was resistant to all 19. Therefore, the capacity to restrict the non-Cercopithecine retroviruses (N-MLV, B-MLV, MPMV, FIV, EIAV, and HIV-1) is an ancestral and conserved property of all Cercopithecine TRIM5αs, and most importantly, restriction of these retroviruses was not determined by the presence or the absence of the V1:G, V1:+2 and V1:G+20 adaptations in V1 (in other words, adding or removing these specific modifications from V1 did not alter the restriction of any non-cercopithecine retrovirus tested, regardless of context).

In contrast, viruses with CA domains from cercopithecine SIVs were only restricted by the subset of TRIM5-V1 variants bearing lineage-specific adaptations in V1 (Fig 2). Specifically, there was a clear correlation between restriction of the eight cercopithecine SIVs in our panel and the presence of those adaptive changes found exclusively in the TRIM5 V1-patch of cercopithecine primates. That these specific adaptions in V1 are sufficient for restriction of cercopithecine viruses is demonstrated by the gain of SIV restriction by the ancestral TRIM5α proteins modified to carry three adaptive changes (ancTRIM5α^V1:TFP^, ancTRIM5α^V1:G^ and ancTRIM5α^V1:G+20^) compared to the unmodified version (ancTRIM5α^V1:Q^) (Fig 2). Moreover, the patterns of restriction associated with each of the reconstructed ancestral TRIM5α proteins resembled those of the modern TRIM5α orthologs naturally bearing the same adaptations (i.e., the V1:G, V1:+2 and V1:G+20 adaptations) (Fig 2). For example, both ancestral reconstructions and modern cercopithecine TRIM5α orthologs with V1:Q or V1:SFP failed to restrict SIV of cercopithecine hosts, whereas both ancTRIM5α^V1:TFP^ and rhTRIM5α^V1:TFP^ from rhesus macaques gave nearly identical patterns of restriction. Similarly, substituting the Q339 residue with a G in the ancTRIM5α to produce ancTRIM5α^V1:G^ only resulted in a gain of ability to restrict a subset of SIVs that was also restricted by modern orthologs from mustached guenons and Schmidt’s guenons, species which naturally bear a G at the homologous position in V1 (musTRIM5α^V1:G^ and sch2TRIM5α^V1:G^ in Fig 2). Finally, ancTRIM5α with the G and the 20 amino-acid insertion (ancTRIM5α^V1:G+20^) only restricted a subset of SIVs that was also restricted by TRIM5 orthologs cloned from African green monkey cell lines, a species which naturally bears the V1:G+20 modification (referred to as agmCTRIM5α^V1:G+20^ and agmVTRIM5α^V1:G+20^ in Fig 2).

It is also noteworthy that TRIM5αs representing each tribe generally did not restrict viruses from the same tribe. Thus, cercopithecin TRIM5αs did not restrict cercopithecin SIVs and, with the exception the TFP alleles, papionin TRIM5αs did not restrict papionin SIVs. This observation is consistent with the possibility that these viruses have been co-evolving with their respective tribes with little or no inter-tribe transmission or recombination between viruses of the two clades.

The specificity conferred by adaptations unique to TRIM5-V1 of cercopithecine monkeys was demonstrated by three additional observations: first, if a reconstructed ancestral TRIM5α protein failed to restrict a virus, modern TRIM5αs naturally bearing the same adaptations were also unable to restrict that virus; second, not every V1 modification led to SIV restriction; third, restriction was usually not observed when an SIV was tested for restriction by the TRIM5α from its established host, regardless of modifications found in V1 (for example, SIVmac was not restricted by the rhesus TRIM5^V1:TFP^ allele, SIVrcm was not restricted by the red-capped mangabey ortholog, and HIV-1 was not restricted by human TRIM5α) (Fig 2).

Finally, we note that M-PMV was previously reported to be resistant to restriction by TRIM5α cloned from COS cells [67], whereas we found this virus was restricted greater than 100-fold by agmCTRIM5a^V1:G+20^. The explanations for this discrepancy include the possibility that different alleles were cloned from the COS cells, or the fact that the reported restriction was performed in HeLa cells (which are capable of expressing endogenous human TRIM5) whereas our assays were done in TRIM5-null CRFK cells. This result does not affect the overall conclusion that adaptations in V1 uniquely affect restriction of cercopithecine SIVs, and sensitivity or resistance of all other viruses to agmCTRIM5α^V1:G+20^ were in agreement with previously published results [36, 53, 67–70].

### Evolution of capsid recognition by Papionini and Cercopithecini TRIM5α involved the same or overlapping targets

All of the naturally occurring TRIM5α orthologs with V1:TFP, V1:G or V1:G+20 adaptions restricted at least three different SIVs (SIVsmE543, SIVstm, SIVrcm) (Fig 2). ancTRIM5α^V1:TFP^, ancTRIM5α^V1:G^ and ancTRIM5α^V1:G+20^ restricted SIVsmE543 and SIVrcm (Fig 2). Taken together, these observations suggest that these TRIM5α variants may recognize the same or similar target(s) in the capsids of these viruses.

We next sought to determine which regions of the CA protein determined the resistant or sensitive phenotypes. To do this, we assayed the ability of our TRIM5-expressing cell lines to restrict a previously described panel of HIV-SIV chimeric viruses in which sequence(s) from the CA domain of HIV-1nl4.3 were introduced into the CA domain of SIVmac239; this panel was successfully used to map determinants of rhesus TRIM5α specificity [25]. We chose 10 TRIM5α variants from species in the subfamily Cercopithecinae to test against this panel of viral mutants in order to identify gain-of-sensitivity CA mutations; 9 of these restrict HIV-1 but not SIVmac239, and a 10^th^, wlfTRIM5α^V1:Q^, does not restrict either HIV-1 or SIVmac239, and was included as a control.

We have previously reported that surface features of CA (β-hairpin, 4–5 loop, helix-6 and the 6–7 loop) largely govern sensitivity/resistance to rhesus TRIM5α alleles, and that the TRIM5α sensitive phenotype can be transferred between HIV-1 and SIVmac239 by the exchange of these features [25]. Similarly, to establish whether surface features of capsid are the primary determinants of restriction by our diverse panel of TRIM5 proteins we assayed restriction of four viruses: these included the two parental viruses (HIV-1nl4.3, 2 and SIVmac239); a modified SIVmac239 virus bearing the CA surface features of HIV-1 (SIV-HIV_surface_); and a modified HIV-1nl4.3 virus bearing the CA surface features of SIVmac239 (HIV-SIV_surface25_) (S5 and S6 Figs) [25]. For the nine TRIM5αs that differentially restrict HIV-1 and SIVmac239 we found that the restricted phenotype was governed by the CA surface and that these phenotypes could be exchanged between viruses by swapping the surface CA features (S8 Fig and S2 Dataset). As predicted the 10^th^ TRIM5α, wlfTRIM5α^V1:Q^, did not restrict the parental viruses or the CA-chimeric viruses.

**Fig 3:**
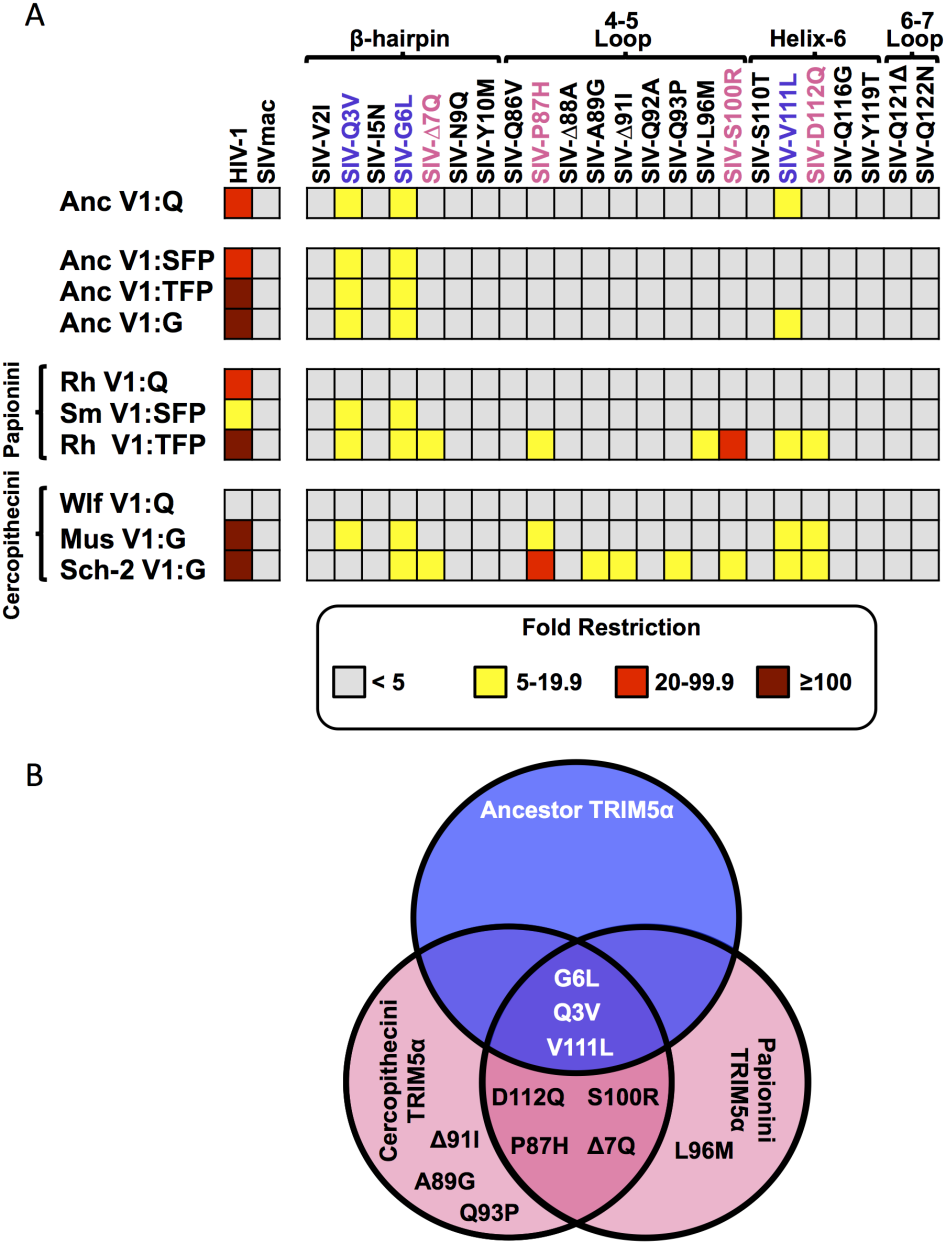
Convergent evolution in capsid targeting among Old World monkey TRIM5αs. A. The indicated TRIM5αs were screened against a panel of HIV-1, SIVmac239 and SIVmac239 viruses with single amino acid substitutions on the CA surface. A key relating the mutation to the position in SIVmac CA and HIV-1 CA is found in Table 2. Extant TRIM5αs are separated by tribe (Papionini and Cercopithecini). Values above 100-fold are given as >100, reflecting the limitations of sensitivity of the FACS assay. Values for each data point can be found in S2 Dataset. B. A Venn diagram showing the overlap between mutations resulting in sensitivity to ancestral TRIM5α-V1:Q and cercopithecin and papionin TRIM5αs.

There are 25 amino acid differences between the CA proteins of the parental HIV-1nl4.3 and HIV-SIV_surface25_ (the chimeric virus in which the CA surface features are derived from SIVmac239). To identify specific sites that modulate the TRIM5α-sensitive phenotype, we next tested a series of SIVmac239 variants in which the amino acid at each of these 25 positions was substituted with the amino acid found at the corresponding position in HIV-1nl4.3 (Table 2) [25]. Of these viruses, 23 were infectious and were assayed for gain-of-sensitivity to restriction by the 10 TRIM5αs (Fig 3A, S5 and S6 Figs).

**Table 2:** Relationship between mutant virus numbering and numbering of SIVmac239 and HIV-1nl4.3. Numbering of mutated residues corresponding to the SIVmac239 CA (Accession number M33262) and HIV-1nl4.3 CA (Accession number M19921.2) are also provided.

Mutant virus	SIVmac239 Residue	HIV-1nl4.3 Residue
V2I	V2	I2
Q3V	Q3	3V
I5N	I5	N5
G6L	G6	L6
Δ7Q	Δ	Q7
N9Q	N8	Q9
Y10M	Y9	M10
Q86V	Q85	V86
P87H	P86	H87
Δ88A	Δ	A88
A89G	A87	G89
Δ91I	Δ	I91
Q92A	Q89	A92
Q93P	Q90	P93
L96M	L93	M96
S100R	S97	R100
S110T	S107	T110
V111L	V108	L111
D112Q	D109	Q112
Q116G	Q113	G116
Y119T	Y116	T119
R120H	R117	H120
R120N	R117	H120
Q121Δ	Q118	Δ
Q122N	Q119	N121
N123P	N120	P122

Interestingly, we observed that the ancestral TRIM5α proteins with the V1:G, V1:+2 and V1:G+20 adaptations did not restrict as many of the SIVmac239-derived capsid mutants as modern TRIM5αs with the same V1 features (Fig 3A). This may indicate that restriction specificity is influenced by multiple determinants in capsid and/or other sites within TRIM5α (including co-evolving sites) such that effects of single point mutations on a particular TRIM5 may be context dependent.

We identified three CA mutations that were individually sufficient to make the SIVmac239 CA sensitive to ancTRIM5α^V1:Q^-mediated restriction (SIVmac239_Q3V_, SIVmac239_G6L_ and SIVmac239_V111L_). As expected, none of the CA mutations resulted in a gain of restriction by wlfTRIM5α^V1:Q^ (which did not restrict either of the parental viruses) (Figs 2 and 3A). Six of the eight remaining TRIM5α proteins also restricted the SIVmac239_Q3V_ and SIVmac239_G6L_ mutant viruses, and a seventh, sch2TRIM5α^G^, restricted SIVmac239_G6L_ but not SIVmac239_Q3V_ (Fig 3A and S2 Dataset). Both of these mutations (Q3V and G6L) map to the β-hairpin, a structural feature that is conserved among the CA proteins of orthoretroviruses [71–76]. AncTRIM5α^V1:Q^ and four additional TRIM5α variants restricted the SIVmac239_V111L_ mutant, which has a substitution in helix-6 (Fig 3A).

Consistent with the notion that modern day TRIM5αs evolved from ancTRIM5α^V1:Q^, we found that extant TRIM5αs largely maintained the capacity to restrict the same SIVmac239 CA-mutant viruses as ancTRIM5α^V1:Q^ (Fig 3A). Many of these extant TRIM5αs restricted a unique subset of the SIVmac239 mutants, indicating that adaptions which alter capsid recognition occurred over the 11+ million years separating the extant TRIM5αs from ancTRIM5α^V1:Q^. We found that only TRIM5αs which restricted cercopithecine SIVs (Fig 2) were capable of restricting more mutant SIVmac239 viruses than ancTRIM5α^V1:Q^. There were four CA mutations, (Δ7Q, P87H, S100R, D112Q) that were restricted by at least one Cercopithecini TRIM5α and one Papionini TRIM5α. For example, the P87H and D112Q mutants were restricted by musTRIM5α^V1:G^, sch2TRIM5α^V1:G^ and rhTRIM5α^V1:TFP^ (Fig 3A). Similarly, both rhTRIM5α^V1:TFP^ and sch2TRIM5α^V1:G^ restricted SIVmac239_Δ7Q_, and SIVmac239_S100R_ (Fig 3). Thus, these two TRIM5αs, from two different Cercopithecine tribes, had highly similar patterns of restriction (Fig 3). The simplest explanation for this observation is that specific Papionini and Cercopithecini TRIM5α orthologs have independently evolved to target Cercopithecine SIVs by targeting similar CA features.

## Discussion

The TRIM5 proteins of primates have a collective capacity to recognize and restrict highly divergent retroviruses from multiple genera, and indeed, some individual orthologs can restrict multiple, distinct retroviruses [77]. Molecular evolutionary analysis also reveals that positive selection, measured as dN/dS ratios, varies significantly in timing and intensity between branches of the primate phylogenetic tree [36], indicating that TRIM5 can evolve at different times in response to the viruses uniquely encountered by different host lineages. Thus, correlating lineage-specific patterns of evolution with specificity for particular types of viruses can provide insight into past virus-host relationships [21]. However, attributing past selective events to a specific type of virus is difficult for several reasons—first, because of positive selection, the phylogenetic tree of a restriction factor may not faithfully recapitulate host phylogeny; second, the viruses responsible for selection may have no extant relatives known to science; and third, serial bouts of selection by different viral agents can alter or obscure the effects of prior adaptations in the restriction factor locus.

We reasoned that the TRIM5 gene of cercopithecine primates should reflect selection due to the emergence of the subset of primate lentiviruses whose descendants are currently endemic to many African monkeys of the Cercopithecinae subfamily. Our analysis was aided by the fact that the phylogenetic relationships of Old World primates are very well established, and by the existence of SIV sequences and isolates from multiple cercopithecine hosts. We identified a small subset of adaptations that arose exclusively in Old World primates of the Cercopithecinae subfamily lineage (including both tribe Papionini and tribe Cercopithecini monkeys), centered on position 339 in V1 (numbering is based on accession NM_001032910.1 as a reference). This includes a Q-to-G substitution at position 339 itself and two independent insertion events at or immediately adjacent to position 339, as well as some subsequent, lineage-specific substitutions that occurred within these inserted sequences. Based on the established phylogenetic relationships among Old World primates, we estimate that these adaptations in V1 began to appear between 11 and 16 million years ago (Fig 1 and Table 1). In stark contrast, the V1 regions of the TRIM5 proteins of all the other Old World primates (Hominoidea species and Colobinae species) are of uniform length, and retain the conserved, ancestral Q at position 339.

Using a reconstructed ancestral TRIM5 protein engineered to contain these cercopithecine-specific adaptations in V1, we show that the changes affect only restriction of extant lentiviruses (SIVs) of cercopithecine monkeys, but do not affect restriction of other lentiviruses, or of retroviruses representing three additional retroviral genera. Likewise, among extant, naturally occurring TRIM5α orthologs, only those containing identical or similar adaptive changes in V1 consistently restricted cercopithecine lentiviruses. In other words, restriction of all other viruses tested was independent of the presence or absence of these adaptations in V1, demonstrating that V1 adaptations unique to the cercopithecine TRIM5 locus were most likely selected by viruses closely related to the SIVs currently endemic to these hosts (the exceptions occur when both the virus and TRIM5 represent the same host species, reflecting host-specific adaptation). While these changes affect restriction of cercopithecine SIVs, it is interesting that they did not affect restriction of other sensitive viruses, such as MLV, which are known to be affected by sequences in the V1 loop [38, 43, 49–51, 78, 79]. Thus, these specific changes resulting in gain-of-specificity for cercopithecine lentiviruses did not overwrite or alter the ability of the PRYSPRY domain to interact with the capsids of the other retroviruses tested.

As mentioned before, a Q at position 339 (or its homologs) in TRIM5 V1 reflects the ancestral state of all catarrhine primates, which last shared a common ancestor ~24–34 million years ago (Table 1). While in non-cercopithecine lineages the ancestral Q has remained unmodified during ~24–34 million years of primate evolution, this position was twice modified by evolution in two independent cercopithecine lineages within a span of approximately 1–4 million years (Fig 1 and Table 1). A reconstructed ancestral TRIM5α representing the last common ancestor of all Old World primates has been reported, and its ability to restrict gammaretroviruses and lentiviruses assayed [80]. Like our somewhat younger (11–16 million year old) ancTRIM5α^V1:Q^ (Fig 2 and S1 Dataset), this ancestor also has a Q in the V1-patch, and like our ancTRIM5α^V1:Q^, this much “older” variant also restricted N-MLV and HIV-1, weakly restricted HIV-2 (~2–4 fold), and did not restrict B-MLV, SIVmac239 or SIVagmTan [80]. This report, along with our results, strengthens the conclusion that adaptations in the V1-loop in the cercopithecine lineage arose in response to a virus or viruses related to the modern SIVs found in these species.

The most parsimonious explanation for our observations is that adaptations in V1 that specifically affect restriction of SIVs from cercopithecine hosts, but not of other retroviruses, reflects selection by lentiviruses related to the modern cercopithecine SIVs. This conclusion is consistent with other observations regarding the prehistory of the lentiviruses. For example, distinct cercopithecine SIV lineages are believed to have existed prior to the isolation of Bioko Island from the African continent at least 10,000 years ago [20], and endogenous lentivirus sequences in the genomes of several mammalian species indicate that viruses related to extant lentiviruses existed at least 3–15 million years ago [4–6, 8, 9].

Evidence for ancient lentiviral infection in the ancestors of Old World primates also comes from the study of patterns of selection in genes with known anti-viral activity, such as tetherin (BST-2) and the APOBEC3 enzymes, and their interactions with viral antagonists of these factors, such as the Nef, Vpu and Vif accessory proteins of modern lentiviruses [21, 22, 81, 82]. Our results lend strong support to these studies by virtue of extending the conclusions to a third, unrelated restriction factor gene (*TRIM5*), which operates via a distinct mechanism and targets a different stage in the retroviral replication cycle. Furthermore, because we determined that adaptations found exclusively in the *TRIM5* genes of cercopithecine species affect only restriction of those lentiviruses naturally found in cercopithecine hosts, but had little effect (either positive or negative) on restriction of any other retrovirus tested (including other primate and non-primate lentiviruses, as well as retroviruses of other genera), we can also extend the results of previous studies by concluding that adaptations in cercopithecine TRIM5 were selected by lentiviruses closely related to the subset of simian immunodeficiency viruses currently found in modern cercopithecine monkeys.

Our observations also suggest that the TRIM5αs representing species in the Papionini and Cercopithecini tribes have independently evolved to restrict endemic lentiviruses through recognition of common or closely overlapping sites on the CA protein, suggesting convergent evolution to target the same feature(s) of the lentiviral capsid core (Fig 3). Specifically, we found that the restriction-sensitive and restriction-resistant phenotypes are largely determined by the CA surface features and that a handful of single amino-acid substitutions within these surfaces are sufficient to render a once resistant virus sensitive (Fig 3 and S8 Fig, S2 Dataset). We identified seven such capsid mutations that affected restriction by both papionin and cercopithecin TRIM5αs, of which four specifically affected restriction by modern papionin and cercopithecin TRIM5αs but not ancTRIM5α^V1:Q^ (Fig 3). Importantly, cercopithecine TRIM5αs which retained the ancestral V1:Q did not restrict any of the four mutant viruses. The fact that the TRIM5αs from Papionini and Cercopithecini monkeys that restricted a common subset of extant cercopithecine SIVs were also similarly affected by the same single-amino acid substitutions in capsid argues that TRIM5α from both tribes recognize similar or overlapping features in CA.

**Fig 4:**
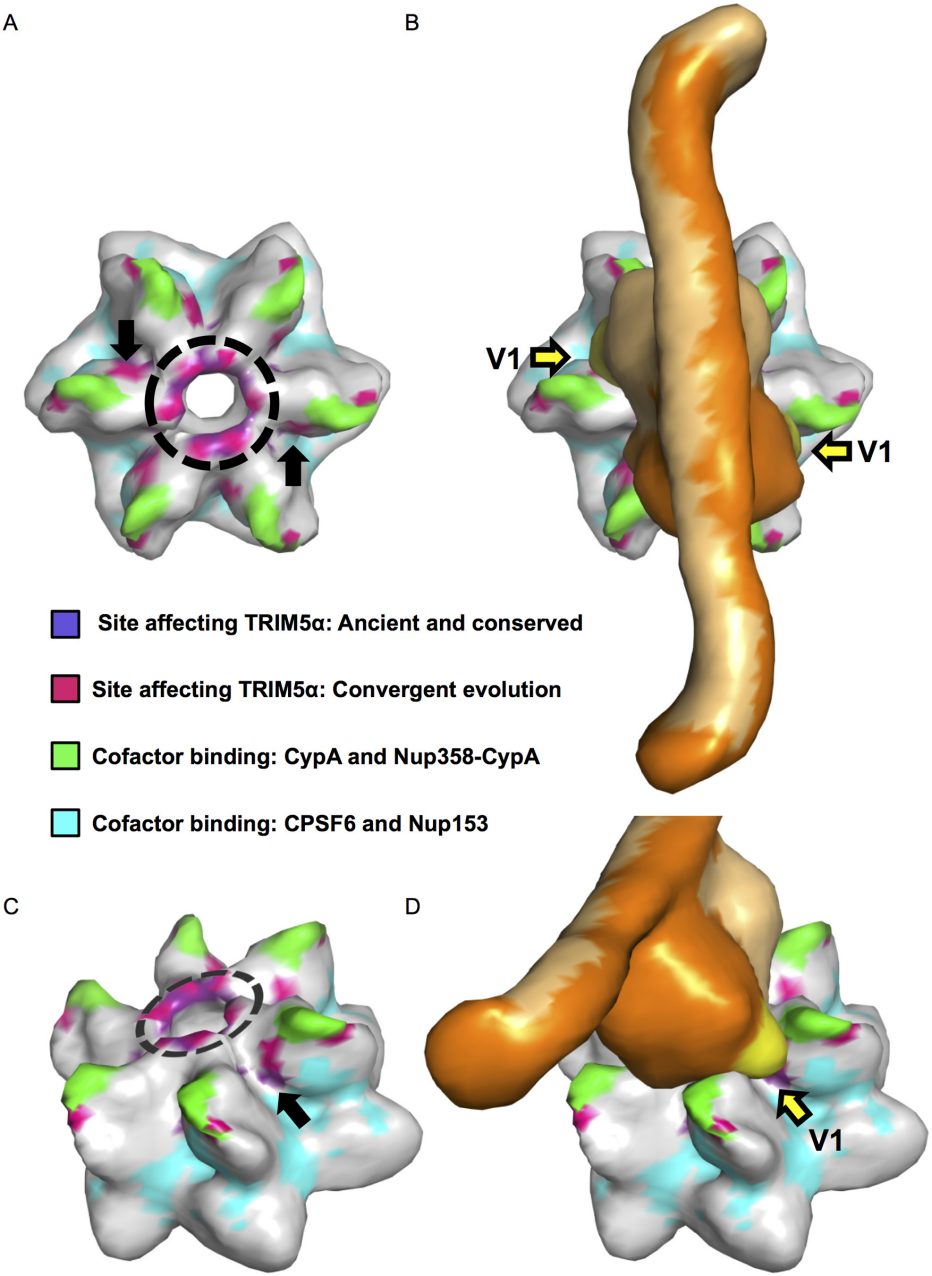
An evolutionarily-guided model for TRIM5α binding to capsid hexamers. A. A capsid hexamer colored according to the key to highlight the sites identified in this study that modulate sensitivity to both Papionini and Cercopithecini TRIM5αs and their relation to sites that mediate contacts with cellular cofactors. A dashed circle shows the position of the β-hairpins. Black arrows point to a cluster of sensitizing mutations that sit between sites that mediate contacts with cyclophilin A/Nup-358-cypA-like domain, and those that mediate contacts with CPSF6/Nup-153. B. A model of TRIM5α (shades of orange) placed on the capsid hexamer. The V1 loops are colored yellow and indicated by yellow arrows. C and D are side views of A and B respectively. This model is based on a TRIM5 model published in Goldstone *et al*., 2014 [92] and provided by Ian Taylor.

The locations in CA of the mutations that sensitize SIVmac239 to papionin and cercopithecin TRIM5α-mediated restriction may be significant. These map to two different regions of the CA protein. The first is the β-hairpin (mutants Q3V, G6L and Δ7Q), which is a structural feature conserved across *Orthoretrovirinae* retrovirus capsids. Mutations at two of these sites, Q3V and G6L, were independently sufficient to render the SIVmac239 capsid sensitive to nearly all of the TRIM5αs assayed, including those that otherwise did not restrict SIVs (Figs 2 and 3). This observation is in agreement with previous proposals that the β-hairpin represents a conserved feature of all retrovirus capsids that is widely exploited by catarrhine TRIM5α proteins [25, 78, 79]. The second set of mutations (V111L, P87H, S100R, and D112Q) cluster in a region responsible for mediating important interactions with host cofactors (S9 Fig). Intriguingly, P87H, S100R and D112Q only affected restriction by the subset of TRIM5α proteins with Cercopithecinae-specific adaptations in V1 (Figs 2, 3 and 4, S9 Fig). Specifically, these sites are found at the base of the 4–5 loop, which for some lentiviruses mediates contacts with Cyclophilin A and Nup-358 [83–85], and are also directly above the binding pocket for CPSF6 and Nup-153 (Fig 4 and S9 Fig) [86–89]. Together, interactions with these cofactors facilitate efficient nuclear import of the viral genome, and are thought to shield the reverse transcription complex from innate immune sensors [84–88, 90, 91]. The broader implication of these observations is that papionin and cercopithecin TRIM5αs may have both adapted to the emergence of lentiviruses by exploiting critical, lentivirus-specific interactions with host-encoded cellular cofactors.

It is plausible that these two regions of CA- the β-hairpin and the junction between cofactor binding sites constitute genuine sites of interaction between TRIM5α and CA. Structures of the TRIM5 region encompassing the B-box and coiled-coil domains indicate that this part of the protein exists as an anti-parallel dimer [92, 93]. Using overlapping residues between this structure [92] and a structure of the PRYSPRY domain [94], a model of the B-box-coiled-coil-PRYSPRY dimer has been generated [92]. This model suggests that the PRYSPRY domains are tucked under the coiled-coils in an arrangement that would position the two PRYSPRY domains such that their variable loops extend in opposite directions [35, 92]. If this model is correct (and barring large-scale conformational rearrangements of TRIM5α upon CA binding), the TRIM5 binding site(s) would also be predicted to face in opposite directions. When mapped onto a HIV-1 CA hexamer, the sites we identified that modulate sensitivity to papionin and cercopithecin TRIM5α proteins are consistent with this prediction, with a spacing that is in general agreement with the published B-box-coiled-coil-PRYSPRY model (Fig 4). Remarkably, when this TRIM5 model is placed over a two-fold axis of symmetry at the center of the CA hexamer, TRIM5 variable loops 2 and 3 sit above the β-hairpins and V1 is oriented towards the junction between cofactor binding sites (Fig 4). While we cannot exclude alternative models, our findings are consistent with models in which TRIM5α engages lentiviral CA through two sets of contacts, one in the structurally conserved β-hairpin and the second at the junction between binding sites of at least four cellular cofactors [25, 78, 79]. Confirmation or rejection of this model will ultimately require structural determination of the PRYSPRY domain in complex with its cognate capsid target.

## Materials and Methods

### Cell lines

TRIM5α variants were isolated from: African green monkey kidney cell lines COS-1 and Vero were obtained from the American Type Culture Collection (Manassas, VA) and grown in DMEM/10% FBS. Skin fibroblast cell lines derived from the following primate species were obtained through Coriell Cell Repositories (Camden, NJ) and cultured according to specification: *Cercocebus torquatus* (red-capped mangabey, PR00485), *Cercopithecus cephus* (mustached guenon, PR00531), *Cercopithecus ascanius* (black-cheeked white-nosed monkey PR00566 and PR00634), *Cercopithecus neglectus* (De Brazza's monkey, PR01144), *Cercopithecus wolfi* (Wolf's guenon PR00486, PR00530 and PR01241).

Crandell-Rees feline kidney (CRFK) cells and human embryonic kidney 293T/17 (HEK293T/17) cells were obtained from American Type Culture Collection (Manassas, VA) and grown in DMEM/10% FBS. CRFK cell lines stably expressing N-terminally HA-tagged TRIM5 orthologs were maintained in DMEM/10% FBS supplemented with 5 μg/ml Puromycin.

### RNA isolation and TRIM5 amplification

RNA was extracted using Trizol reagent (Ambion/Life Technologies). cDNA was prepared using a Transcriptor First Strand cDNA Synthesis Kit (Roche) using an anchored-oligo(dT)_18_ primer. TRIM5α cDNAs were amplified and N-terminally HA-tagged using TRIM5-F-GCGGAATTCGCCACCATGTACCCATACGACGTCCCAGACTACGCTGGCGGCGCTTCTGGAATCCTGCTTAATGTAAAG AND TRIM5-R-ACCATCGATGGCTCAAGAGCTTGGTGAGCACAGAGTC primers. PCR amplicons were directly coloned into pLPCX (Clonetech) using EcoRI and ClaI sites.

### Plasmids

TRIM5αs were cloned into pLPCX using EcoRI and ClaI sites. Retroviral GFP-reporter viruses were produced from the following plasmids: HIV-1 was produced from the following reagent that was obtained through the NIH AIDS Reagent Program, Division of AIDS, NIAID, NIH: pNL4-3-deltaE-EGFP (Cat# 11100) from Drs. Haili Zhang, Yan Zhou, and Robert Siliciano [95]. The pV1EGFP derivatives encoding the 5’ region of SIVmac239, SIVsmE543, SIVstm/37.16 were previously described [96]. N-tropic or B-tropic MLVs from either pCIG-N or pCIG-B and pLXIN-EGFP (gifts of Jonathan Stoye, Medical Research Council, London, U.K.). Rous sarcoma virus (RSV) [97] (Addgene plasmid 13878, courtesy of Constance Cepko, Harvard Medical School, MA), Equine infectious anemia virus (EIAV) pEV53D and pEIAV-SIN6.1 CGFPW (Addgene plasmids 44168 and 44171 courtesy of John Olsen, University of North Carolina) [98, 99]. Feline immunodeficiency virus (FIV) pFP93 and pGINSIN (gifts from Eric Poeschla, Mayo Clinic) [100, 101]. The first 205 amino acids of the pNL4-3-deltaE-EGFP CA were replaced with the equivalent stretch of the following SIV CAs similar to previously published reports [102, 103]: SIVrcm (AF349680), SIVagmVerv (L40990), SIVagmGrv (M66437) and SIVmus-1 (AY340700). These CA were synthesized as Strings by GeneArt/Life Technologies and cloned into pNL4-3-deltaE-EGFP using a previously described shuttle vector [25]. HIV-1nl4.3-SIVmac239 chimeric viruses were previously described [25].

### Virus production

All single-cycle viruses were produced in HEK293T/17 cells by cotransfection of the appropriate viral plasmid(s) and pVSV-G (Clontech Laboratories, Mountain View, CA), using GenJet (SignaGen; Ijamsville, MD). Viral supernatants were titered on CRFK cells; supernatant volumes resulting in approximately 25% GFP/EGFP+ CRFK cells were used for infectivity assays on the cell lines expressing the indicated ortholog of TRIM5α.

### Infectivity assays

Stably expressing TRIM5 CRFK cells were seeded at a concentration of 5×10^4^ cells per well in 24-well-plates and infected with the appropriate amount of VSV-G pseudotyped, single-cycle, GFP/EGFP expressing viruses. After 2 days, expression of GFP/EGFP was analyzed by fluorescence-activated cell sorting (FACS) performed on a FACSCaliburTM flow cytometer (BD, Franklin Lakes, NJ), and data were analyzed using FlowJo software (Tree Star, Inc., Ashland, OR).

### Phylogenetic reconstruction

To predict a TRIM5α amino-acid sequence representing the last common ancestor of all extant Cercopithecinae species, we generated an alignment of 60 unique catarrhine TRIM5α sequences, including those first reported in this study and those obtained from publicly available databases. A maximum likelihood phylogenetic tree was generated in Geneious (Biomatters Limited, Auckland New Zealand) using the PhyML plugin [104]; the TRIM5α tree topology approximated the established relationships of Old World primates (S2 Fig). This tree and corresponding alignment were used for ancestral node reconstruction via the FASTML server (http://fastml.tau.ac.il/) [64–66]. The sequence of ancTRIM5α^V1:Q^ corresponds to the predicted nodal sequence for the last common ancestor of all cercopithecine TRIM5αs (S3 Fig). A synthetic version of this sequence including a N-terminal HA tag was generated (Genescript, Piscataway, NJ) and subcloned into pLPCX (Clonetech Laboratories, Mtn. View, CA). The 339Q/G substitution and the 2 amino-acid insertions were generated by mutagenic PCR using this plasmid as a template. The 20 amino-acid insertion was ordered as part of smaller fragment (Genescript, Piscataway, NJ) and subcloned into the ancTRIM5α^V1:Q^ vector to create ancTRIM5α^V1:G+20^. Constructs were then used to generate stable cell lines as described.

### Western blotting

Cells were lysed in M-PER reagent (Pierce Biotechnology, Rockford, IL) and mixed with an equal volume of 2x Laemmli sample buffer (Sigma, St. Louis, MO) and solubilized by boiling for 10 min at 99°C. Protein was separated by SDS/PAGE. β-actin was detected using a mouse monoclonal antibody (20272) (Abcam, Cambridge England). HA was detected with a rabbit polyclonal sera (PA1-29751) (Pierce Biotechnology, Rockford, IL) using dilutions recommended by the manufacturer. HIV-1 p17 was detected using the following reagent which was obtained through the NIH AIDS Reagent Program, Division of AIDS, NIAID, NIH: Anti-HIV-1 p17 Polyclonal (VU47) from Dr. Paul Spearman [105].

### Accession numbers

The GenBank accession numbers for TRIM5αs sequenced for this study are KP743973-KP743978.

## Supporting Information

S1 Fig Proposed order of steps for the evolution of the two amino acid insertion. A Q at position 339 was overwritten by a two amino acid insertion in an ancestor common to all extant Papionini species and has continued to evolve. A. A sequence alignment of the region immediately around the V1-patch of Papionini TRIM5αs. Nucleotide and amino acid differences are colored red. B. A phylogenetic tree of Papionini species. Major events that have altered the sequence of the V1-patch have been mapped onto this tree. C. The nucleotide substitutions that have led to Papionini V1-patch diversity. Underlined regions indicate sites that underwent changes. Arrows show what modified sites became in subsequent intermediates. Red text indicates sites that have changed from one line to the next. We propose that the initial insertion event was due to the duplication of two codons corresponding to rhesus TRIM5α positions 338 and 339 (FQ). Residues 339–341 would then encode for QFQ. Single nucleotide substitutions in codons 339 and 341 alter the sequence of 339–341 to encode for QFP or PFQ. A second single nucleotide substitution results in PFP. This variant has been found in baboon TRIM5α sequences. Single amino acid substitutions within codon 339 result in the SFP, TFP, MFP and LFP variants found in nature. We note that it is unclear whether TFP arose from PFP or SFP.
(PDF)

S2 Fig Phylogenetic relationships of TRIM5 sequences of 60 extant Old World monkeys and apes. A maximum likelihood tree was generated in Geneious (Auckland, New Zealand)[104] and subsequently rendered in Figtree (http://tree.bio.ed.ac.uk/software/figtree). The tree was rooted on the node separating Old World monkeys and apes. Taxonomic groups are color-coded. The leaves are labeled with Genbank accession numbers and common names of all species, and the node used for reconstruction of the ancestral TRIM5 sequence is indicated by a black dot. The scale bar indicates substitutions per site.
(PDF)

S3 Fig Sequence of ancTRIM5α^V1:Q^. The following features are annotated: RING domain (black and underlined), linkers (gray), B-box (green and underlined), coiled-coil (purple and underlined), PRYSPRY. The V1 loop is bolded and underlined. The ancestral Q at position 339 is highlighted in blue.
(PDF)

S4 Fig Capsid sequences of HIV-1 viruses engineered to express SIV capsids. A sequence alignment of the capsids that were inserted into HIV-1. Bolded and underlined sequences indicate the HIV-1 sequences that were left at the extreme C-terminus which has been previously reported to improve the infectivity of HIV-1 viruses with substituted capsids [102, 103].
(PDF)

S5 Fig Capsid or Matrix content of mutant viruses. A. Concentrations of p24 or p27 (capsid) for HIV-1nl4.3 SIVmac239, and HIV-1nl4.3-SIVmac239 chimeric viruses. B. A western blot for HIV-1nl4.3 p17 (matrix) showing the relative concentrations and processing of HIV-1 viruses in which the capsid has been substituted with those of other SIVs. 1 ml of virus was pelleted and subjected to Western blotting with anti-HIV-1 p17 sera, VU47 [105]. The HIV-1 pellet was prepared from the same stock as panel A.
(PDF)

S6 Fig Titration curves of mutant viruses. A. HIV-1nl4.3 B. SIVmac239. C. SIV-HIV_surface_. D. HIV-SIV_surface25_. E. SIVmac239_V2I_ F.SIVmac239_Q3V_. G. SIVmac239_I5N_. H. SIVmac239_G6L_. I. SIVmac239_Δ7Q_. J. SIVmac239_N9Q_. K. SIVmac239_Y10M_. L. SIVmac239_Q86V_. M. SIVmac239_P87H_. N. SIVmac239_Δ88A_. O. SIVmac239_A89G_. P. SIVmac239_Δ91I_. Q. SIVmac239_Q92A_. R. SIVmac239_Q93P_. S. SIVmac239_L96M_. T. SIVmac239_S100R_. U. SIVmac239_S110T_. V. SIVmac239_V111L_. W. SIVmac239_D112Q_. X. SIVmac239Q_116G_. Y. SIVmac239_Y119T_. Z. SIVmac239_Q121Δ_. AA. SIVmac239_Q122N_. AB. “SIVrcm” HIV-1-SIVrcm-SCA. AC. “SIVmus” HIV-1-SIVmus-SCA. AD. “SIVagmVer” HIV-1-SIVagmVer-SCA. AE. “SIVagmGrv” HIV-1-SIVagmGrv-SCA. AF. “SIVcol” HIV-1-SIVcol-SCA AG. “SIVdeb” HIV-1-SIVdeb-SCA. AH. “SIVdrl” HIV-1-SIVdrl-SCA. AI. “SIVgsn” HIV-1-SIVgsn-SCA. AJ. “SIVmnd-1” HIV-1-SIVmnd-1-SCA. AK. “SIVmnd-2” HIV-1-SIVmnd-2-SCA.
(PDF)

S7 Fig Expression of HA-tagged TRIM5αs. Cell lysates were subject to Western blotting for HA and β-actin. Lane numbers correspond to: 1. Puromycin control cells 2. ancTRIM5α^V1:Q^ 3. rhTRIM5α^V1:Q^ 4. ancTRIM5α^V1:QFQ^ 5. ancTRIM5α^V1:PFP^ 6. ancTRIM5α^V1:SFP^ 7. rcmTRIM5α^V1:SFP^ 8. smTRIM5α^V1:SFP^ 9. ancTRIM5α^V1:TFP^ 10. rhTRIM5α^V1:TFP^ 11. Puromycin control cells 12. ancTRIM5α^V1:Q^ 13. wlfTRIM5α^V1:Q^ 14. ancTRIM5α^V1:G^ 15. sch1TRIM5α^V1:G^ 16. sch2TRIM5α^V1:G^ 17. musTRIM5α^V1:G^ 18. debTRIM5α^V1:G^ 19. Puromycin control 20. ancTRIM5α^V1:Q^ 21. ancTRIM5α^V1:G+20^ 22. agmVTRIM5α^V1:G+20^ 23. agmCTRIM5α^V1:G+20^ 24. huTRIM5α^V1:Q^
(PDF)

S8 Fig Capsid surface features are the major determinant of Old World monkey TRIM5α restriction. A subset of TRIM5αs that differentially restrict HIV-1nl4.3 and SIVmac239 were identified. We tested these TRIM5αs with chimeric viruses to determine whether the major determinant of this phenotype was the surface of the CA protein. Cell lines were infected with wild type SIVmac239, HIV-1nl4.3, SIV with the HIV-1 surface (SIV-HIV_surface_), and HIV with the SIV surface (HIV-SIV_surface25_). These viruses have been previously described [25] and additional information is provided in S5 and S6 Figs Fold restriction was graphed for each virus. Values above 100-fold are given as >100, reflecting the limitations of sensitivity of the FACS assay. Values for each data point can be found in S2 Dataset.
(PDF)

S9 Fig A. A sequence alignment of N-terminal domains of the primate lentiviruses used in this study. Black residues indicate sites that are 100% conserved between these viruses. HIV-1 residues involved in contacts with cellular cofactors are indicated with colored triangles at the top of the alignment. B. An HIV-1 capsid structure with the 100% conserved sites colored black. C. An HIV-1 capsid structure with CPSF6 interacting residues colored light blue. D. An HIV-1 capsid structure with Nup-153 interacting residues colored dark blue. E. An HIV-1 capsid structure with cyclophillin interacting residues colored light green. F. Sites that modulate TRIM5 sensitivity are colored in purple and pink.
(PDF)

S1 Dataset Supporting data for Fig 2. The fold restriction and standard error for each data point in Fig 2. Restriction is abbreviated as “rxn”. Each data point is the average of at least three independent experiments. Values above 100 fold are given as >100, reflecting the limitations of sensitivity of the FACS assay.
(PDF)

S2 Dataset Supporting data for Fig 3 and S8 Fig. The fold restriction and standard error for each data point in Fig 3. Restriction is abbreviated as “rxn”. Each data point is the average of at least three independent experiments. Values above 100 fold are given as >100, reflecting the limitations of sensitivity of the FACS assay.
(PDF)
