# Supplementary material for: Evolutionary and Functional Analysis of Old World Primate TRIM5 Reveals the Ancient Emergence of Primate Lentiviruses and Convergent Evolution Targeting a Conserved Capsid Interface
Source: PLoS Pathog. 2015 Aug 20;11(8):e1005085. doi: 10.1371/journal.ppat.1005085 (PMC4546234; doi:10.1371/journal.ppat.1005085)
Supplement: S1 Fig — A Q at position 339 was overwritten by a two amino acid insertion in an ancestor common to all extant Papionini species and has continued to evolve. A. A sequence alignment of the region immediately around the V1-patch of Papionini TRIM5αs. Nucleotide and amino acid differences are colored red. B. A phylogenetic tree of Papionini species. Major events that have altered the sequence of the V1-patch have been mapped onto this tree. C. The nucleotide substitutions that have led to Papionini V1-patch diversity. Underlined regions indicate sites that underwent changes. Arrows show what modified sites became in subsequent intermediates. Red text indicates sites that have changed from one line to the next. We propose that the initial insertion event was due to the duplication of two codons corresponding to rhesus TRIM5α positions 338 and 339 (FQ). Residues 339–341 would then encode for QFQ. Single nucleotide substitutions in codons 339 and 341 alter the sequence of 339–341 to encode for QFP or PFQ. A second single nucleotide substitution results in PFP. This variant has been found in baboon TRIM5α sequences. Single amino acid substitutions within codon 339 result in the SFP, TFP, MFP and LFP variants found in nature. We note that it is unclear whether TFP arose from PFP or SFP. (PDF) [file ppat.1005085.s001.pdf]

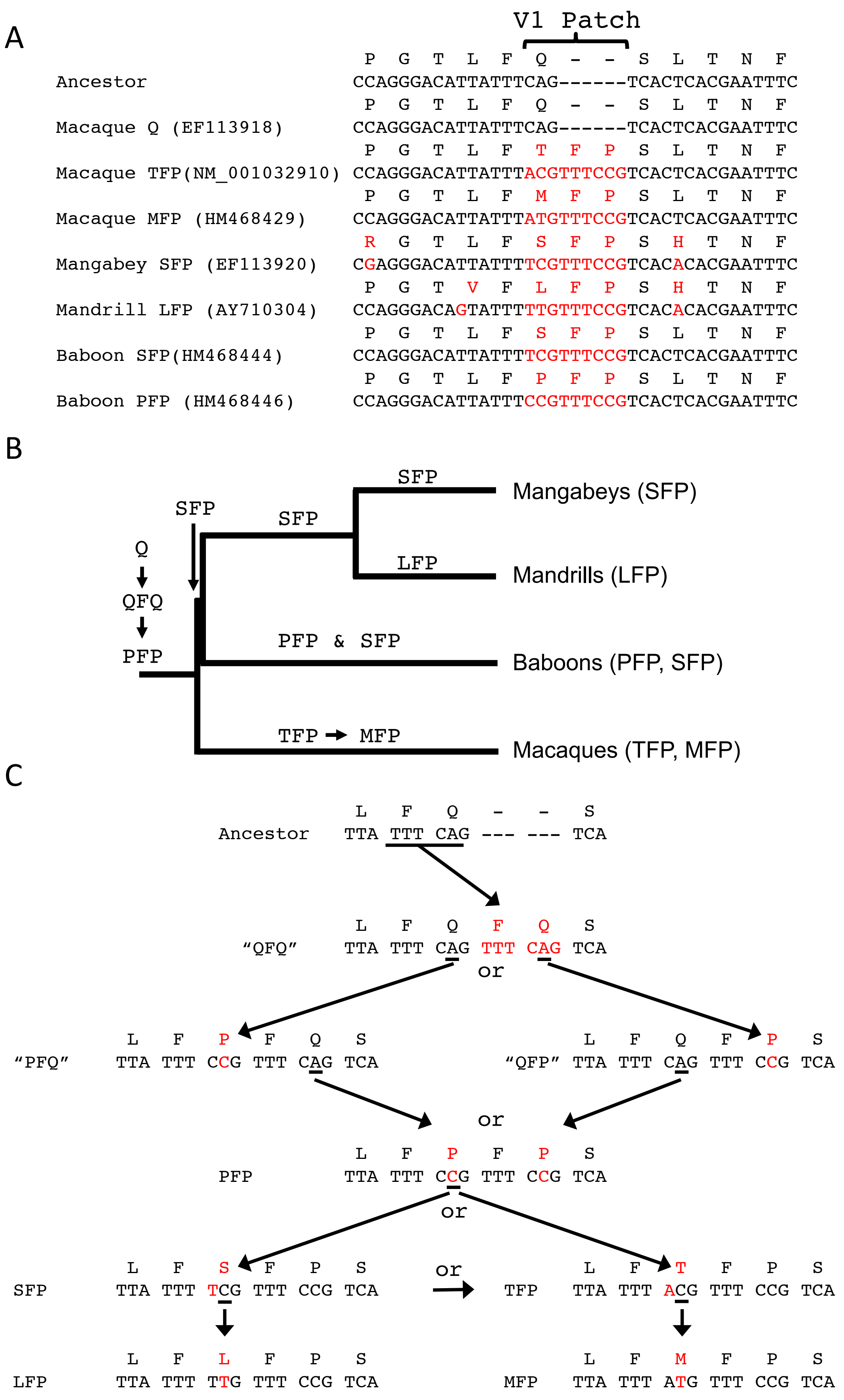
A
V1 Patch
P G T L F Q - - S L T N F
Ancestor CCAGGGACATTATTTCAG------TCACTCACGAATTTC
P G T L F Q - - S L T N F
Macaque Q (EF113918) CCAGGGACATTATTTCAG------TCACTCACGAATTTC
P G T L F T F P S L T N F
Macaque TFP(NM_001032910) CCAGGGACATTATTTACGTTTCCGTCACTCACGAATTTC
P G T L F M F P S L T N F
Macaque MFP (HM468429) CCAGGGACATTATTTATGTTTCCGTCACTCACGAATTTC
R G T L F S F P S H T N F
Mangabey SFP (EF113920) CGAGGGACATTATTTTCGTTTCCGTCACACACGAATTTC
P G T V F L F P S H T N F
Mandrill LFP (AY710304) CCAGGGACAGTATTTTTGTTTCCGTCACACACGAATTTC
P G T L F S F P S L T N F
Baboon SFP(HM468444) CCAGGGACATTATTTTCGTTTCCGTCACTCACGAATTTC
P G T L F P F P S L T N F
Baboon PFP (HM468446) CCAGGGACATTATTTCCGTTTCCGTCACTCACGAATTTC
B
SFP
Mangabeys (SFP)
SFP
SFP
Q
QFQ
LFP
Mandrills (LFP)
PFP
PFP & SFP
Baboons (PFP, SFP)
TFP → MFP
Macaques (TFP, MFP)
C
L F Q - - S
Ancestor TTA TTT CAG --- --- TCA
L F Q F Q S
"QFQ" TTA TTT CAG TTT CAG TCA
or
L F P F Q S
"PFQ" TTA TTT CCG TTT CAG TCA
L F Q F P S
"QFP" TTA TTT CAG TTT CCG TCA
or
L F P F P S
PFP TTA TTT CCG TTT CCG TCA
or
L F S F P S
SFP TTA TTT TCG TTT CCG TCA
or
TFP
L F T F P S
TTA TTT ACG TTT CCG TCA
L F L F P S
LFP TTA TTT TTG TTT CCG TCA
L F M F P S
MFP TTA TTT ATG TTT CCG TCA
