# Supplementary figures and images for: Evolutionary and Functional Analysis of Old World Primate TRIM5 Reveals the Ancient Emergence of Primate Lentiviruses and Convergent Evolution Targeting a Conserved Capsid Interface
Source: PLoS Pathog. 2015 Aug 20;11(8):e1005085. doi: 10.1371/journal.ppat.1005085 (PMC4546234; doi:10.1371/journal.ppat.1005085)

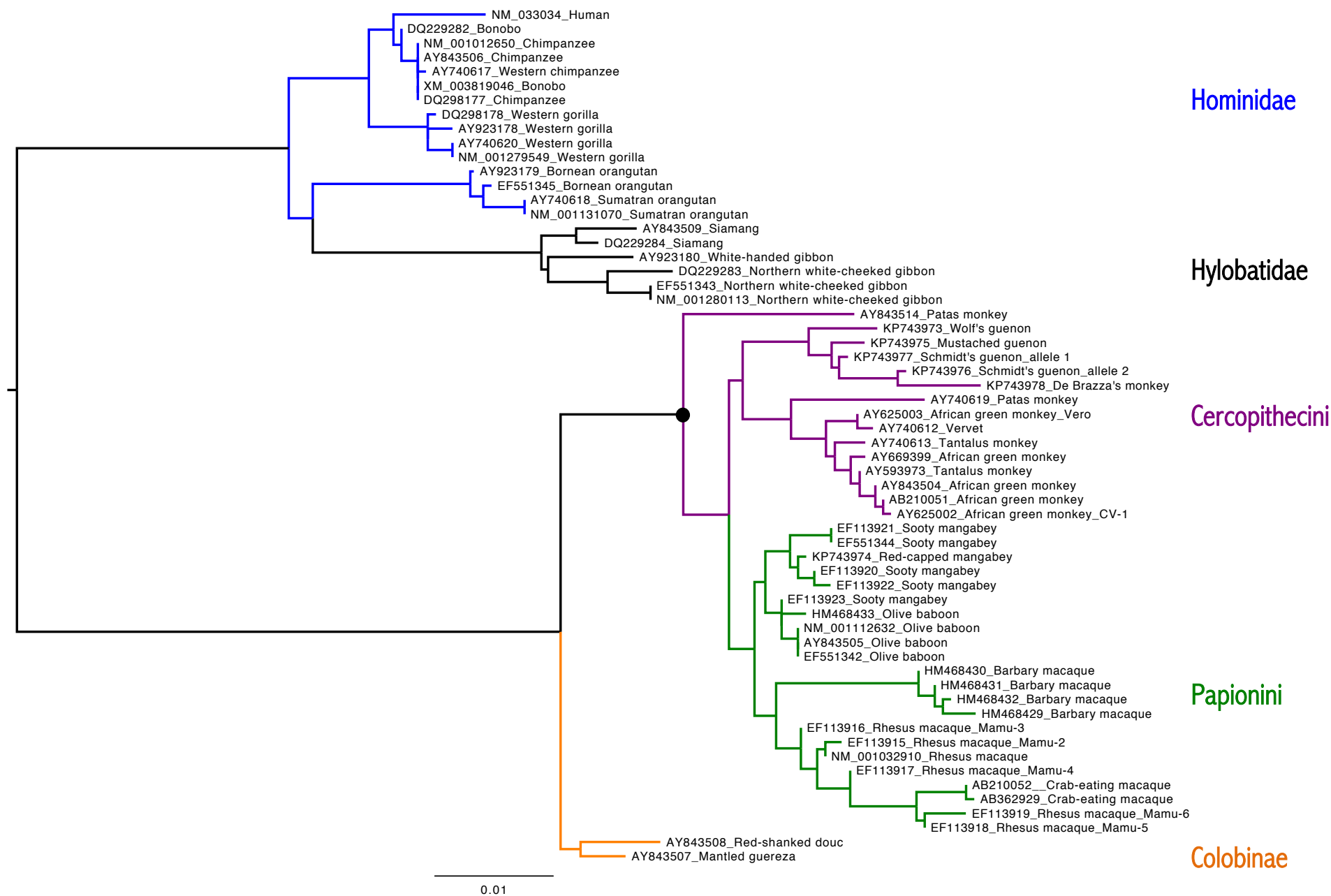

Supplement: S2 Fig — A maximum likelihood tree was generated in Geneious (Auckland, New Zealand)[104] and subsequently rendered in Figtree (http://tree.bio.ed.ac.uk/software/figtree). The tree was rooted on the node separating Old World monkeys and apes. Taxonomic groups are color-coded. The leaves are labeled with Genbank accession numbers and common names of all species, and the node used for reconstruction of the ancestral TRIM5 sequence is indicated by a black dot. The scale bar indicates substitutions per site. (PDF) [file ppat.1005085.s002.pdf]

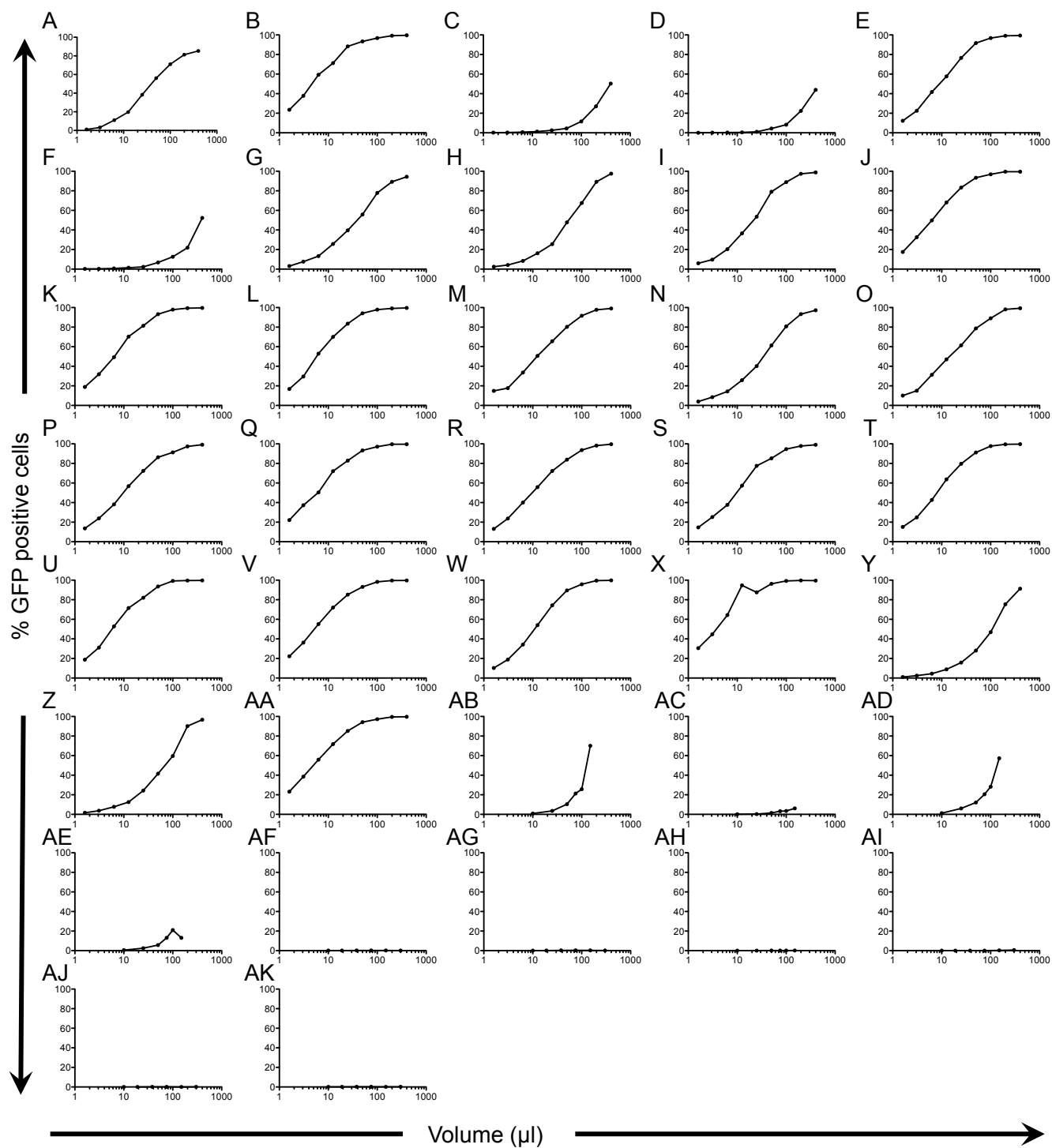

Supplement: S6 Fig — A. HIV-1nl4.3 B. SIVmac239. C. SIV-HIVsurface. D. HIV-SIVsurface25. E. SIVmac239V2I F.SIVmac239Q3V. G. SIVmac239I5N. H. SIVmac239G6L. I. SIVmac239Δ7Q. J. SIVmac239N9Q. K. SIVmac239Y10M. L. SIVmac239Q86V. M. SIVmac239P87H. N. SIVmac239Δ88A. O. SIVmac239A89G. P. SIVmac239Δ91I. Q. SIVmac239Q92A. R. SIVmac239Q93P. S. SIVmac239L96M. T. SIVmac239S100R. U. SIVmac239S110T. V. SIVmac239V111L. W. SIVmac239D112Q. X. SIVmac239Q116G. Y. SIVmac239Y119T. Z. SIVmac239Q121Δ. AA. SIVmac239Q122N. AB. “SIVrcm” HIV-1-SIVrcm-SCA. AC. “SIVmus” HIV-1-SIVmus-SCA. AD. “SIVagmVer” HIV-1-SIVagmVer-SCA. AE. “SIVagmGrv” HIV-1-SIVagmGrv-SCA. AF. “SIVcol” HIV-1-SIVcol-SCA AG. “SIVdeb” HIV-1-SIVdeb-SCA. AH. “SIVdrl” HIV-1-SIVdrl-SCA. AI. “SIVgsn” HIV-1-SIVgsn-SCA. AJ. “SIVmnd-1” HIV-1-SIVmnd-1-SCA. AK. “SIVmnd-2” HIV-1-SIVmnd-2-SCA. (PDF) [file ppat.1005085.s006.pdf]

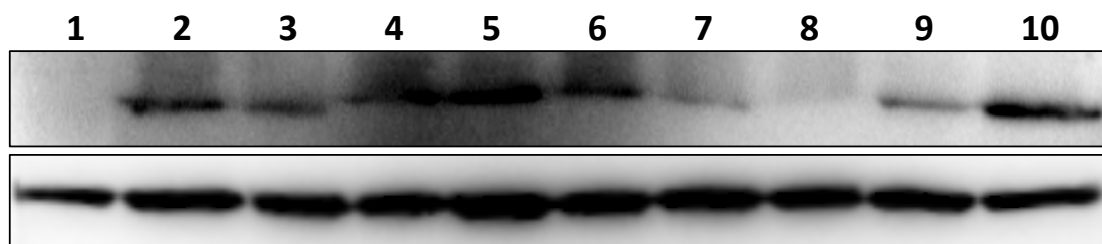

IB: HA

IB:  $\beta$ -actin

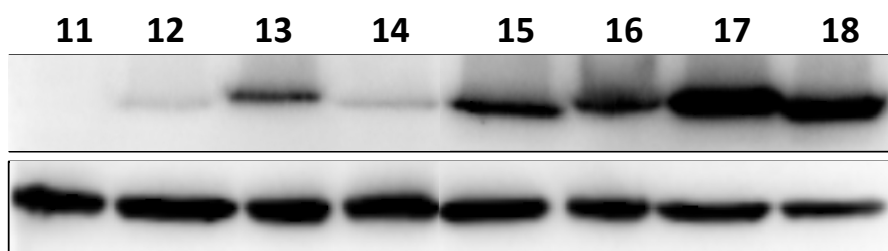

IB: HA

IB:  $\beta$ -actin

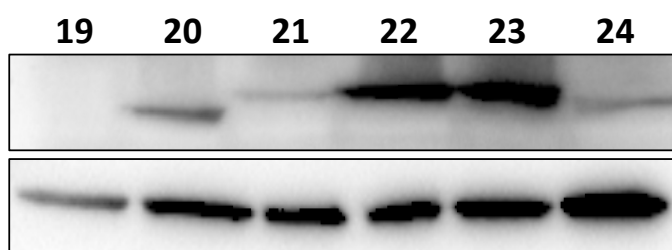

IB: HA

IB:  $\beta$ -actin

Supplement: S7 Fig — Cell lysates were subject to Western blotting for HA and β-actin. Lane numbers correspond to: 1. Puromycin control cells 2. ancTRIM5αV1:Q 3. rhTRIM5αV1:Q 4. ancTRIM5αV1:QFQ 5. ancTRIM5αV1:PFP 6. ancTRIM5αV1:SFP 7. rcmTRIM5αV1:SFP 8. smTRIM5αV1:SFP 9. ancTRIM5αV1:TFP 10. rhTRIM5αV1:TFP 11. Puromycin control cells 12. ancTRIM5αV1:Q 13. wlfTRIM5αV1:Q 14. ancTRIM5αV1:G 15. sch1TRIM5αV1:G 16. sch2TRIM5αV1:G 17. musTRIM5αV1:G 18. debTRIM5αV1:G 19. Puromycin control 20. ancTRIM5αV1:Q 21. ancTRIM5αV1:G+20 22. agmVTRIM5αV1:G+20 23. agmCTRIM5αV1:G+20 24. huTRIM5αV1:Q (PDF) [file ppat.1005085.s007.pdf]

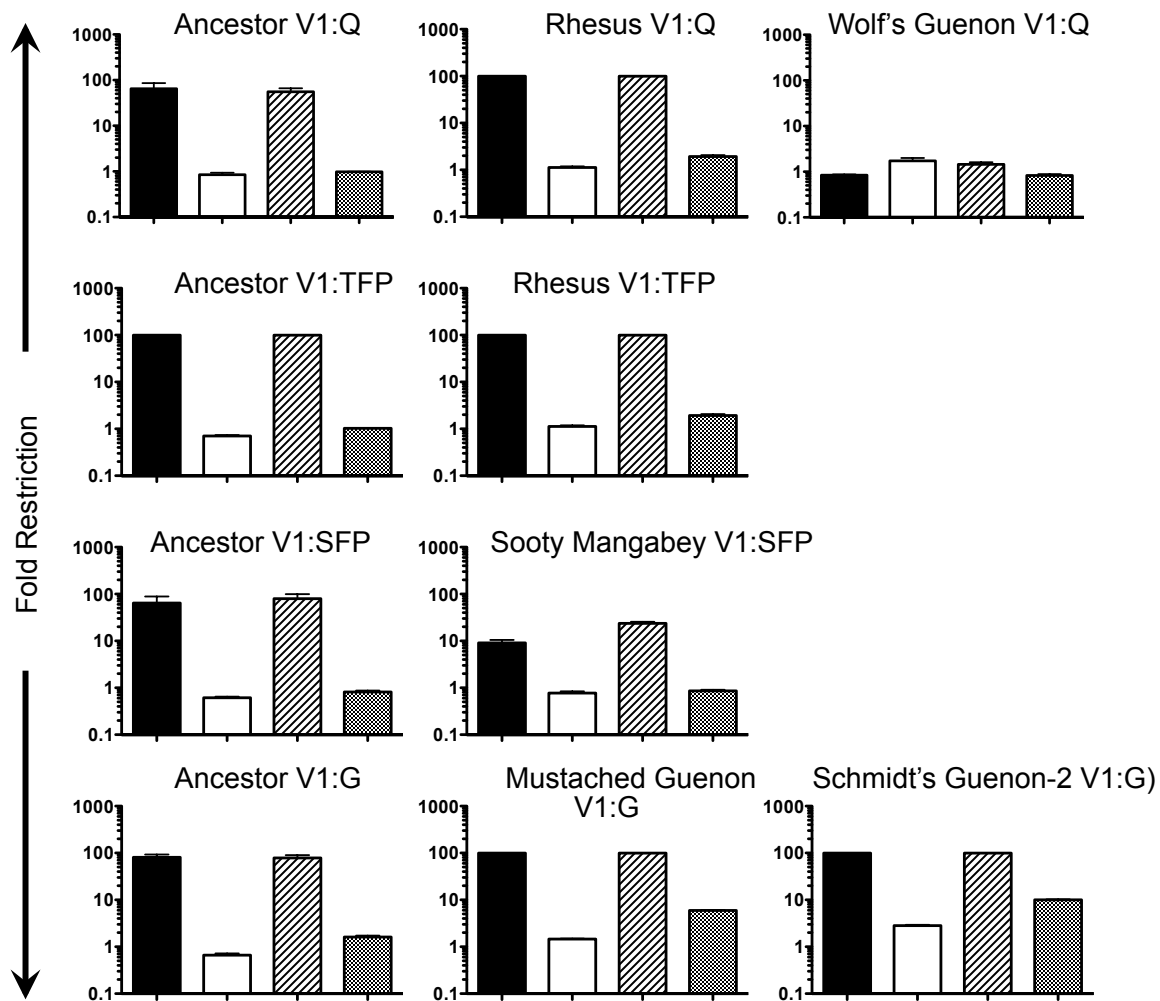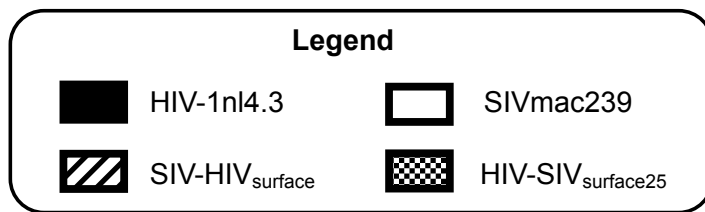

Supplement: S8 Fig — A subset of TRIM5αs that differentially restrict HIV-1nl4.3 and SIVmac239 were identified. We tested these TRIM5αs with chimeric viruses to determine whether the major determinant of this phenotype was the surface of the CA protein. Cell lines were infected with wild type SIVmac239, HIV-1nl4.3, SIV with the HIV-1 surface (SIV-HIVsurface), and HIV with the SIV surface (HIV-SIVsurface25). These viruses have been previously described [25] and additional information is provided in S5 and S6 Figs Fold restriction was graphed for each virus. Values above 100-fold are given as >100, reflecting the limitations of sensitivity of the FACS assay. Values for each data point can be found in S2 Dataset. (PDF) [file ppat.1005085.s008.pdf]
