# Supplementary material for: Evolutionary and Functional Analysis of Old World Primate TRIM5 Reveals the Ancient Emergence of Primate Lentiviruses and Convergent Evolution Targeting a Conserved Capsid Interface
Source: PLoS Pathog. 2015 Aug 20;11(8):e1005085. doi: 10.1371/journal.ppat.1005085 (PMC4546234; doi:10.1371/journal.ppat.1005085)
Supplement: S3 Fig — The following features are annotated: RING domain (black and underlined), linkers (gray), B-box (green and underlined), coiled-coil (purple and underlined), PRYSPRY. The V1 loop is bolded and underlined. The ancestral Q at position 339 is highlighted in blue. (PDF) [file ppat.1005085.s003.pdf]

MASGILVNVKEEVTCPICLELLTEPLSLPCGHSFCQACITANHKKSMLYKEGERSCPVCRIS
YQPENIRPNRHVANIVEKLREVKLSPEEGQKVDHCARHGEKLLLFCQEDSKVICWLCERS
QEHRGHHTFLMEEVAQEYHVKLQTALEMLRQKQQEAEKLEADIREEKASWKIQIDYDKT
NVSADFEQLREILDWEESNELQNLEKEEEDILKSLTKSETEMVQQTQYMRELISDLEHRL
QGSMMELLQGVDGIIKRIENMTLKKPKTFHKNQRRVFRAPDLKGMLDMFRELTDVRRY
WVDVTLAPNNISHAVIAEDKRQV**SSRNPQIMYQAPGTLFQSLTNFNYCTGVLGS**QSITS
GKHYWEVDVSKKSAWILGVCAGFQPDAMYNIEQNENYQPKYGYWVIGLQEGVKYSVFQD
GSSHTPFAPFIVPLSVIICPDRVGVFVDYEACTVSFFNITNHGFLIYKFSQCSFSKPVFPYLNP
RKCTVPMTLCSPSS
