# Supplementary material for: Evolutionary and Functional Analysis of Old World Primate TRIM5 Reveals the Ancient Emergence of Primate Lentiviruses and Convergent Evolution Targeting a Conserved Capsid Interface
Source: PLoS Pathog. 2015 Aug 20;11(8):e1005085. doi: 10.1371/journal.ppat.1005085 (PMC4546234; doi:10.1371/journal.ppat.1005085)
Supplement: S4 Fig — A sequence alignment of the capsids that were inserted into HIV-1. Bolded and underlined sequences indicate the HIV-1 sequences that were left at the extreme C-terminus which has been previously reported to improve the infectivity of HIV-1 viruses with substituted capsids [102, 103]. (PDF) [file ppat.1005085.s004.pdf]

>HIV-1nl4.3
PIVQNLQGQMVHQAISPRTLNAWVKVVEEKAFSPEVIPMFSALSEGATPQDLNTMLNTVGGHQAAMQMLKETINEEAAEWDRLHPVHAGPIAPGQMREP
RGSDIAGTTSTLQEQIGWMTHN-----
PPIPVGEIYKRWIILGLNKIVRMYSPTSILDIRQGPKEPFRDYVDRFYKTLRAEQASQEVKNWMTETLLVQNANPDCKTILKAL**GPGATLEEMMTACQG**
VGGPGHKARVL
>HIV-SIVrcm-sca
PIVTIN-QQPEHQPISPRTLNAWVKVVEEKKFGAEVVPMFSALSEGCIPYDVNQMLNAIGEHQGALQIVKEVINDEAADWDLRHPNP-
GPLPAGQLREPTGSDIAGTTSNIAEQIAWTTRA----
NNPIAVGNIYRNWIVLGLQKCVKMYNPVNILDIKQGPKEPFKDYVDRFYKALRAEQADPAVKNWMTQSLLIQNANPDCKTVLKGL**GPGATLEEMMTACQ**
GVGGPGHKARVL
>HIV-SIVmus-sca
PVIRNAQGQFQHQALNARILKTWVSIVEEKKFAAEVVAMFQALAEGAIPYDINQLLNAIGEHQGAIQIIKDVINEQAAEWDLLNPQPQQPQPNAGLRNP
TGSDIAGVSSTPQEQIEWITRA----
NNPINVAEIYKKWVIMGLQRCVKMYNPVNILDIKQGPKEPFKDYVDRFFKCLRAEQSDQAVKNWMTSTLLVQNANPECKLILKSM**GPGATLEEMMTACQ**
GVGGPGHKARVL
>HIV-SIVagmVer-sca
PAQQQG-
NAWVHVPLSPRTLNAWVKAVEEKKFGAEIVPMFQALSEGCTPYDINQMLNVLGDHQGALQIVKEIINEEAAQWDVTHPPPAGPLPPGQLRDPRGSDIAG
TTSTVQEQLEWIYTA----
NPRVDVGAIYRRWIILGLQKCVKMYNPVSVLDIRQGPKEPFKDYVDRFYKAIRAEQASGEVKQWMTESLLIQNANPDCKVILKGL**GPGATLEEMMTACQ**
GVGGPGHKARVL
>HIV-SIVagmGRV-sca
PVVNQN-
NAWVHQPLSPRTLNAWVKCVEEKRWGAEVVPMFQALSEGCLSYDVNQMLNVIGDHQGALQILKEVINEEAAEWDRTHRPPAGPLPAGQLRDPTGSDIAG
TTSSIQEQIEWTFNA----
NPRIDVGAQYRKWVILGLQKVVQMYNPQKVLDIRQGPKEPFQDYVDRFYKALRAEQAPQDVKNWMTQTLLIQNANPDCKLILKGL**GPGATLEEMMTACQ**
GVGGPGHKARVL
>HIV-SIVdeb-sca
PILRQG-QQFVHMPLSPRIVKTWINAVEEKKFSPEIVPLFQVLAEGCTPYDINGLLNAIGDLQGAMQIIKDVINEEAAEWDLQHPQQ-QP-
PQGQLREPSGADIAGTNSTVEEQIAWMTRPAGQGQGPIDVGQIYRRWVILGLQRCVKMYNPTNILDVKQGPKEPFKDYVDRFYKTLRAEQADQAVKNWM
TTTLMIQNANPDCRIILKGL**GPGATLEEMMTACQGVGGPGHKARVL**
>HIV-SIVdrl-sca
PIQVVN-QQAVHQAISPRTLNAWVKVIEEKKFSAEVVPMFLALSEGCISYDINQMLNAIGDHQGALQIVKDIINEEAADWDRRHPQV-
GPLPQGVLRNPSGSDIAGTTSSIEEQIEWTTRA----
QDSINVGAIYRQWVVLGLQRCVTMYNPVNILDVKQGPKEPFKDYVDRFYKALRAEHTDAAVKNWMTQTLLIQNANPDCKVILKGL**GPGATLEEMMTACQ**
GVGGPGHKARVL
>HIV-SIVgsn-sca
PIVRNAQGQFQHQALNSRVLKTWVSIVEEKKFAPETVALFQALTEGCIPYDMNQMLNAVGDYQGAVQIIKDVINEQAAEWDLLHPQPAAPQPVAGLRDP
SGADIAGVTSTPNEQIEWITRQ----
NNPVNVADIYRKWIILGLQRCVKMYNPVNILGIKQGPKEPFKEYVDRFFKCLRAEQADQAVKNWMTQLLLVQNANPECKLILKAM**GPGATLEEMMTACQ**
GVGGPGHKARVL
>HIV-SIVmnd-1-sca
PVQRDAAGQYQYTPISPRIIQTWVKTVEEKKWKPEVIPLFSALTEGAISHDLNIMLNAVGDHQGAMQVLKDVINEQAAEWDLTHPQQQPAQPGGGLRTP
SGSDIAGTTSTVEEQLAWMNMQ----
QNAINVGTIYKSWIILGMNRLVKSHCPISITDVRQGPKEAFKDYVDRFYNVMRAEQASGEVKMWMQQHLLIENANPECKQILRSL**GPGATLEEMMTACQ**
GVGGPGHKARVL
>HIV-SIVmdd-2-sca
PIQIIN-QTPVHQGISPRTLNAWVKCIEEKKFSPEIVPMFIALSEGCLPYDLNGMLNAIGEHQGALQIVKDVINEEAADWDLRHPQV-
GPLPQGVLRNPTGSDIAGTTSSIEEQIEWTTRQ----
QEQVNVGAIYKQWIVLGLQKCVSMYNPVNILDIKQGPKEPFKDYVDRFYKALRAERTDPQVKTWMTQTLLIQNANPDCKSILKGL**GPGATLEEMMTACQ**
GVGGPGHKARVL
>HIV-SIVsab-sca
PIVSVN-
NQWVHQPLSPRTLNAWVKVIEEKKFSAEVVPMFSALAEGAIPYDINQMLNAVGEHQGALQIVKDVINEEAADWDLRHPPPQQPPAQGVLRDPQGSDIAG
TTSTIQEQIEWTTRA----
QNAVNVGNIYKGWIILGLQKCVKMYNPVNILDIKQGPKEPFKDYVDRFYKALRAEQTDPAVKNWMTQSLLIQNANPDCKTVLKGL**GPGATLEEMMTACQ**
GVGGPGHKARVL
>HIV-SIVcol-sca
PIVTGP-QGPVHQPLSPRTLGAWVKCVE-GGIAPSLAPMFLAYSTGAIAYDMNLMLNILDTHQGFPQVLKDEINKKAEEYDLLHPVQ-
QPQQQGALRQPTASDITGNTSSVAEQVAWGEPI----------
ANIYRGWIVQSLEKVIQIARPSSVLDIRQGSKEDFKSYVDRFYSALRAEPAAGEIKAWMANNLLIQHANPDCKRILKGL**GPGATLEEMMTACQGVGGPG**
HKARVL
