# Supplementary material for: Evolutionary and Functional Analysis of Old World Primate TRIM5 Reveals the Ancient Emergence of Primate Lentiviruses and Convergent Evolution Targeting a Conserved Capsid Interface
Source: PLoS Pathog. 2015 Aug 20;11(8):e1005085. doi: 10.1371/journal.ppat.1005085 (PMC4546234; doi:10.1371/journal.ppat.1005085)

A

Virus	Capsid Production µg/ml
HIV-1nl4.3	2.81
SIVmac239	2.06
SIV-HIVsurface	0.15
HIV-SIVsurface25	0.96
SIV-V2I	1.1
SIV-Q3V	1.01
SIV-I5N	1.39
SIV-G6L	0.8
SIV-∆7Q	0.54
SIV-N9Q	1.38
SIV-Y10M	2.1
SIV-Q86V	1.9
SIV-P87H	1.6
SIV-∆88A	1.55
SIV-A89G	2.15
SIV-∆91I	2.01
SIV-Q92A	3.48
SIV-Q93P	5.46
SIV-L96M	3.58
SIV-S100R	1.93
SIV-S110T	1.94
SIV-V111L	2.11
SIV-D112Q	1.16
SIV-Q116G	2.69
SIV-Y119T	0.89
SIV-Q121∆	1.22
SIV-Q122N	3.2

B

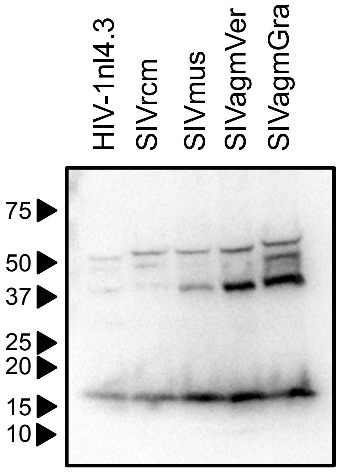

Supplement: S5 Fig — A. Concentrations of p24 or p27 (capsid) for HIV-1nl4.3 SIVmac239, and HIV-1nl4.3-SIVmac239 chimeric viruses. B. A western blot for HIV-1nl4.3 p17 (matrix) showing the relative concentrations and processing of HIV-1 viruses in which the capsid has been substituted with those of other SIVs. 1 ml of virus was pelleted and subjected to Western blotting with anti-HIV-1 p17 sera, VU47 [105]. The HIV-1 pellet was prepared from the same stock as panel A. (PDF) [file ppat.1005085.s005.pdf]
