# Supplementary material for: Evolutionary and Functional Analysis of Old World Primate TRIM5 Reveals the Ancient Emergence of Primate Lentiviruses and Convergent Evolution Targeting a Conserved Capsid Interface
Source: PLoS Pathog. 2015 Aug 20;11(8):e1005085. doi: 10.1371/journal.ppat.1005085 (PMC4546234; doi:10.1371/journal.ppat.1005085)
Supplement: S9 Fig — Black residues indicate sites that are 100% conserved between these viruses. HIV-1 residues involved in contacts with cellular cofactors are indicated with colored triangles at the top of the alignment. B. An HIV-1 capsid structure with the 100% conserved sites colored black. C. An HIV-1 capsid structure with CPSF6 interacting residues colored light blue. D. An HIV-1 capsid structure with Nup-153 interacting residues colored dark blue. E. An HIV-1 capsid structure with cyclophillin interacting residues colored light green. F. Sites that modulate TRIM5 sensitivity are colored in purple and pink. (PDF) [file ppat.1005085.s009.pdf]

A

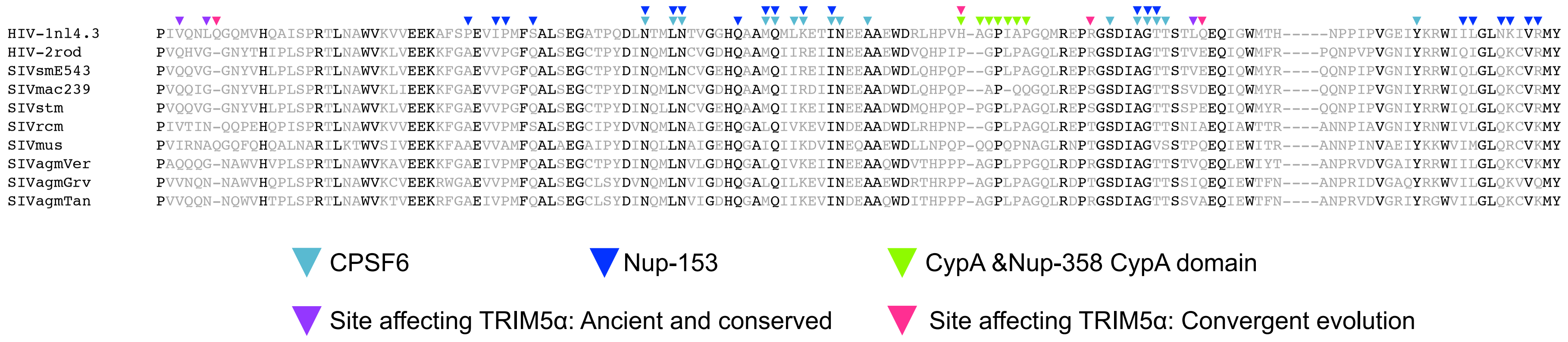
HIV-1nl4.3 PIVQNLQGQMVHQAISPRTLNAWVKVVEEKAFSPEVIPMFSALSEGATPQDLNTMLNTVGGHQAAMQMLKETINEEAAEWDRLHPVH-AGPIAPGQMREPRGSDIAGTTSTLQEQIGWMTH-----NPPIPVGEIYKRWIILGLNKIVRMY
HIV-2rod PVQHVG-GNYTHIPLSPRTLNAWVKLVEEKKFGAEVVPGFQALSEGCTPYDINQMLNCVGDHQAAMQIIREIINEEAAEWDVQHPIP--GPLPAGQLREPRGSDIAGTTSTVEEQIQWMFR----PQNPVPVGNIYRRWIQIGLQKCVRMY
SIVsmE543 PVQQVG-GNYVHLPLSPRTLNAWVKLVEEKKFGAEVVPGFQALSEGCTPYDINQMLNCVGEHQAAMQIIREIINEEAADWDLQHPQP--GPLPAGQLREPRGSDIAGTTSTVEEQIQWMYR----QQNPIPVGNIYRRWIQLGLQKCVRMY
SIVmac239 PVQQIG-GNYVHLPLSPRTLNAWVKLIEEKKFGAEVVPGFQALSEGCTPYDINQMLNCVGDHQAAMQIIRDIINEEAADWDLQHPQP--AP-QQGQLREPSGSDIAGTTSSVDEQIQWMYR----QQNPIPVGNIYRRWIQLGLQKCVRMY
SIVstm PVQQVG-GNYVHLPLSPRTLNAWVKLVEEKKFGAEVVPGFQALSEGCTPYDINQLLNCVGEHQAAMQIIKEIINEEAADWDMQHPQP-PGPLPAGQLREPSGSDIAGTTSSPEEQIQWMYR----QQNPIPVGNIYRRWIQLGLQKCVRMY
SIVrcm PIVTIN-QQPEHQPISPRTLNAWVKVVEEKKFGAEVVPMFSALSEGCIPYDVNQMLNAIGEHQGALQIVKEVINDEAADWDLRHPNP--GPLPAGQLREPTGSDIAGTTSNIAEQIAWTTR----ANNPIAVGNIYRNWIVLGLQKCVKMY
SIVmus PVIRNAQGQFQHQALNARILKTWVSIVEEKKFAAEVVAMFQALAEGAIPYDINQLLNAIGEHQGAIQIIKDVINEQAAEWDLLNPQP-QQPQPNAGLRNPTGSDIAGVSSTPQEQIEWITR----ANNPINVAEIYKKWVIMGLQRCVKMY
SIVagmVer PAQQQG-NAWVHVPLSPRTLNAWVKAVEEKKFGAEIVPMFQALSEGCTPYDINQMLNVLGDHQGALQIVKEIINEEAAQWDVTHPPP-AGPLPPGQLRDPRGSDIAGTTSTVQEQLEWIYT----ANPRVDVGAIYRRWIILGLQKCVKMY
SIVagmGrv PVVNQN-NAWVHQPLSPRTLNAWVKCVEEKRWGAEVVPMFQALSEGCLSYDVNQMLNVIGDHQGALQILKEVINEEAAEWDRTHRPP-AGPLPAGQLRDPTGSDIAGTTSSIQEQIEWTFN----ANPRIDVGAQYRKWVILGLQKVVQMY
SIVagmTan PVVQQN-NQWVHTPLSPRTLNAWVKTVEEKRFGAEIVPMFQALSEGCLSYDINQMLNVIGDHQGAMQIIKEVINDEAAQWDITHPPP-AGPLPAGQLRDPRGSDIAGTTSSVAEQIEWTFN----ANPRVDVGRIYRGWVILGLQKCVKMY
CPSF6
Nup-153
CypA &Nup-358 CypA domain
Site affecting TRIM5α: Ancient and conserved
Site affecting TRIM5α: Convergent evolution

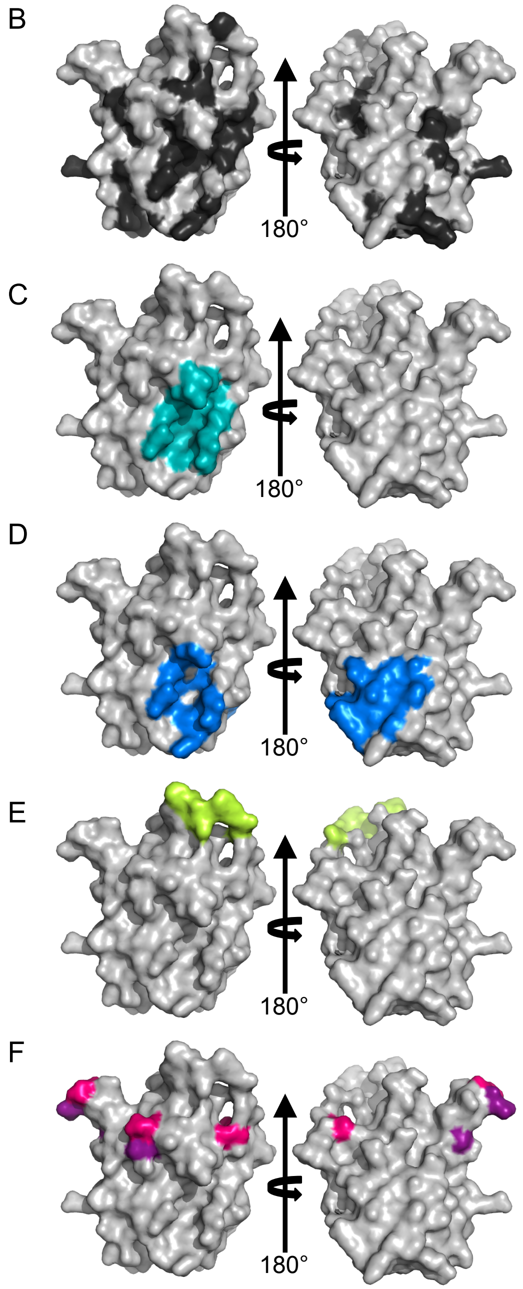

B
180°
C
180°
D
180°
E
180°
F
180°
