# Supplementary material for: Evolutionary and Functional Analysis of Old World Primate TRIM5 Reveals the Ancient Emergence of Primate Lentiviruses and Convergent Evolution Targeting a Conserved Capsid Interface
Source: PLoS Pathog. 2015 Aug 20;11(8):e1005085. doi: 10.1371/journal.ppat.1005085 (PMC4546234; doi:10.1371/journal.ppat.1005085)
Supplement: S1 Dataset — The fold restriction and standard error for each data point in Fig 2. Restriction is abbreviated as “rxn”. Each data point is the average of at least three independent experiments. Values above 100 fold are given as >100, reflecting the limitations of sensitivity of the FACS assay. (PDF) [file ppat.1005085.s010.pdf]

TRIM5	virus	Average fold rxn	SEM
Anc-Q	RSV	0.74	0.08
Anc-Q	MPMV	0.74	0.05
Anc-Q	B-MLV	0.76	0.05
Anc-Q	N-MLV	60.68	10.95
Anc-Q	EIAV	26.81	5.29
Anc-Q	FIV	>100	-
Anc-Q	HIV-1	35.41	12.29
Anc-Q	HIV-2	3.55	0.42
Anc-Q	SIVsmE543	1.04	0.15
Anc-Q	SIVmac239	0.72	0.08
Anc-Q	SIVstm	1.04	0.10
Anc-Q	SIVrcm	0.80	0.20
Anc-Q	SIVmus	0.65	0.16
Anc-Q	SIVagmVerv	0.97	0.13
Anc-Q	SIVagmGrv	0.80	0.07
Anc-Q	SIVagmTan	0.98	0.06

Rhesus (Q)	RSV	0.95	0.16
Rhesus (Q)	MPMV	0.84	0.17
Rhesus (Q)	B-MLV	0.85	0.05
Rhesus (Q)	N-MLV	>100	-
Rhesus (Q)	EIAV	49.00	12.49
Rhesus (Q)	FIV	>100	-
Rhesus (Q)	HIV-1	28.41	2.70
Rhesus (Q)	HIV-2	5.98	0.70
Rhesus (Q)	SIVsmE543	1.24	0.32
Rhesus (Q)	SIVmac239	0.79	0.14
Rhesus (Q)	SIVstm	0.95	0.11
Rhesus (Q)	SIVrcm	0.97	0.31
Rhesus (Q)	SIVmus	0.97	0.34
Rhesus (Q)	SIVagmVerv	1.33	0.40
Rhesus (Q)	SIVagmGrv	0.97	0.05
Rhesus (Q)	SIVagmTan	1.02	0.12

Wolf's (G)	RSV	0.82	0.14
Wolf's (G)	MPMV	1.00	0.31
Wolf's (G)	B-MLV	3.50	1.09
Wolf's (G)	N-MLV	>100	-
Wolf's (G)	EIAV	14.32	1.25
Wolf's (G)	FIV	8.87	0.76
Wolf's (G)	HIV-1	1.73	0.58

Wolf's (G)	HIV-2	0.82	0.06
Wolf's (G)	SIVsmE543	0.77	0.08
Wolf's (G)	SIVmac239	0.77	0.06
Wolf's (G)	SIVstm	0.78	0.05
Wolf's (G)	SIVrcm	0.62	0.14
Wolf's (G)	SIVmus	1.27	0.38
Wolf's (G)	SIVagmVerv	0.79	0.18
Wolf's (G)	SIVagmGrv	1.37	0.48
Wolf's (G)	SIVagmTan	0.83	0.06

Anc-QFQ	RSV	0.77	0.08
Anc-QFQ	MPMV	0.85	0.06
Anc-QFQ	B-MLV	0.73	0.03
Anc-QFQ	N-MLV	33.26	1.88
Anc-QFQ	EIAV	>100	-
Anc-QFQ	FIV	>100	-
Anc-QFQ	HIV-1	25.71	3.12
Anc-QFQ	HIV-2	2.40	0.29
Anc-QFQ	SIVsmE543	0.80	0.07
Anc-QFQ	SIVmac239	0.78	0.08
Anc-QFQ	SIVstm	0.92	0.06
Anc-QFQ	SIVrcm	0.91	0.18
Anc-QFQ	SIVmus	0.84	0.21
Anc-QFQ	SIVagmVerv	1.25	0.21
Anc-QFQ	SIVagmGrv	0.93	0.04
Anc-QFQ	SIVagmTan	0.92	0.04

Anc-PFP	RSV	1.02	0.12
Anc-PFP	MPMV	1.00	0.16
Anc-PFP	B-MLV	0.97	0.12
Anc-PFP	N-MLV	20.05	1.22
Anc-PFP	EIAV	5.31	0.12
Anc-PFP	FIV	36.02	2.79
Anc-PFP	HIV-1	>100	-
Anc-PFP	HIV-2	9.10	0.74
Anc-PFP	SIVsmE543	2.83	0.34
Anc-PFP	SIVmac239	0.80	0.03
Anc-PFP	SIVstm	1.06	0.05
Anc-PFP	SIVrcm	1.26	0.13
Anc-PFP	SIVmus	4.34	2.05
Anc-PFP	SIVagmVerv	1.76	0.42
Anc-PFP	SIVagmGrv	1.51	0.07

Anc-PFP	SIVagmTan	1.02	0.01

Anc-SFP	RSV	1.08	0.07
Anc-SFP	MPMV	0.89	0.07
Anc-SFP	B-MLV	0.97	0.07
Anc-SFP	N-MLV	>100	-
Anc-SFP	EIAV	38.58	2.25
Anc-SFP	FIV	>100	-
Anc-SFP	HIV-1	>100	-
Anc-SFP	HIV-2	7.66	1.75
Anc-SFP	SIVsmE543	1.86	0.14
Anc-SFP	SIVmac239	0.79	0.06
Anc-SFP	SIVstm	0.95	0.06
Anc-SFP	SIVrcm	0.81	0.17
Anc-SFP	SIVmus	0.87	0.27
Anc-SFP	SIVagmVerv	0.88	0.09
Anc-SFP	SIVagmGrv	0.81	0.14
Anc-SFP	SIVagmTan	0.91	0.09

SM (SFP)	RSV	0.94	0.14
SM (SFP)	MPMV	1.54	0.22
SM (SFP)	B-MLV	1.20	0.20
SM (SFP)	N-MLV	>100	-
SM (SFP)	EIAV	50.03	2.71
SM (SFP)	FIV	>100	-
SM (SFP)	HIV-1	13.45	2.95
SM (SFP)	HIV-2	2.03	0.26
SM (SFP)	SIVsmE543	0.89	0.16
SM (SFP)	SIVmac239	0.80	0.09
SM (SFP)	SIVstm	0.95	0.08
SM (SFP)	SIVrcm	0.81	0.16
SM (SFP)	SIVmus	1.07	0.11
SM (SFP)	SIVagmVerv	1.22	0.30
SM (SFP)	SIVagmGrv	0.86	0.11
SM (SFP)	SIVagmTan	0.96	0.09

RCM (SFP)	RSV	0.85	0.17
RCM (SFP)	MPMV	0.65	0.02
RCM (SFP)	B-MLV	0.75	0.07
RCM (SFP)	N-MLV	21.49	0.03
RCM (SFP)	EIAV	17.46	1.69
RCM (SFP)	FIV	73.82	7.00

RCM (SFP)	HIV-1	32.85	6.75
RCM (SFP)	HIV-2	4.58	0.52
RCM (SFP)	SIVsmE543	1.11	0.10
RCM (SFP)	SIVmac239	0.75	0.06
RCM (SFP)	SIVstm	0.83	0.03
RCM (SFP)	SIVrcm	0.63	0.08
RCM (SFP)	SIVmus	1.07	0.12
RCM (SFP)	SIVagmVerv	0.72	0.09
RCM (SFP)	SIVagmGrv	0.95	0.43
RCM (SFP)	SIVagmTan	0.92	0.04

Anc-TFP	RSV	0.75	0.05
Anc-TFP	MPMV	0.86	0.11
Anc-TFP	B-MLV	0.88	0.04
Anc-TFP	N-MLV	10.53	0.22
Anc-TFP	EIAV	10.29	0.59
Anc-TFP	FIV	>100	-
Anc-TFP	HIV-1	>100	-
Anc-TFP	HIV-2	>100	-
Anc-TFP	SIVsmE543	80.61	9.70
Anc-TFP	SIVmac239	0.78	0.05
Anc-TFP	SIVstm	5.59	0.14
Anc-TFP	SIVrcm	7.09	1.05
Anc-TFP	SIVmus	1.38	0.53
Anc-TFP	SIVagmVerv	>100	-
Anc-TFP	SIVagmGrv	25.40	4.51
Anc-TFP	SIVagmTan	12.27	1.52

Rhesus (TFP)	RSV	0.72	0.05
Rhesus (TFP)	MPMV	0.96	0.05
Rhesus (TFP)	B-MLV	0.70	0.06
Rhesus (TFP)	N-MLV	22.25	6.48
Rhesus (TFP)	EIAV	25.59	4.10
Rhesus (TFP)	FIV	>100	-
Rhesus (TFP)	HIV-1	>100	-
Rhesus (TFP)	HIV-2	>100	-
Rhesus (TFP)	SIVsmE543	>100	-
Rhesus (TFP)	SIVmac239	1.05	0.04
Rhesus (TFP)	SIVstm	43.17	4.23
Rhesus (TFP)	SIVrcm	51.87	10.05
Rhesus (TFP)	SIVmus	4.30	0.74
Rhesus (TFP)	SIVagmVerv	>100	-

Rhesus (TFP)	SIVagmGrv	73.19	15.59
Rhesus (TFP)	SIVagmTan	58.28	7.79

Anc-G	RSV	0.94	0.19
Anc-G	MPMV	0.76	0.07
Anc-G	B-MLV	0.86	0.14
Anc-G	N-MLV	88.77	9.86
Anc-G	EIAV	>100	-
Anc-G	FIV	70.86	14.90
Anc-G	HIV-1	>100	-
Anc-G	HIV-2	68.93	18.70
Anc-G	SIVsmE543	5.80	0.97
Anc-G	SIVmac239	0.65	0.06
Anc-G	SIVstm	3.75	0.37
Anc-G	SIVrcm	36.74	6.22
Anc-G	SIVmus	1.95	0.47
Anc-G	SIVagmVerv	1.02	0.10
Anc-G	SIVagmGrv	0.84	0.26
Anc-G	SIVagmTan	0.82	0.07

Mus (G)	RSV	1.27	0.29
Mus (G)	MPMV	0.87	0.18
Mus (G)	B-MLV	0.89	0.08
Mus (G)	N-MLV	>100	-
Mus (G)	EIAV	>100	-
Mus (G)	FIV	>100	-
Mus (G)	HIV-1	>100	-
Mus (G)	HIV-2	>100	-
Mus (G)	SIVsmE543	>100	-
Mus (G)	SIVmac239	1.05	0.19
Mus (G)	SIVstm	24.61	2.11
Mus (G)	SIVrcm	33.04	6.21
Mus (G)	SIVmus	0.91	0.20
Mus (G)	SIVagmVerv	1.36	0.43
Mus (G)	SIVagmGrv	1.23	0.07
Mus (G)	SIVagmTan	0.92	0.10

Schmidt-2 (G)	RSV	0.98	0.08
Schmidt-2 (G)	MPMV	0.93	0.15
Schmidt-2 (G)	B-MLV	0.88	0.07
Schmidt-2 (G)	N-MLV	>100	-
Schmidt-2 (G)	EIAV	>100	-

Schmidt-2 (G)	FIV	>100	-
Schmidt-2 (G)	HIV-1	>100	-
Schmidt-2 (G)	HIV-2	>100	-
Schmidt-2 (G)	SIVsmE543	>100	-
Schmidt-2 (G)	SIVmac239	2.09	0.43
Schmidt-2 (G)	SIVstm	47.31	8.60
Schmidt-2 (G)	SIVrcm	47.63	6.66
Schmidt-2 (G)	SIVmus	1.28	0.16
Schmidt-2 (G)	SIVagmVerv	1.40	0.35
Schmidt-2 (G)	SIVagmGrv	1.69	0.17
Schmidt-2 (G)	SIVagmTan	1.03	0.08

Schmidt-1 (G)	RSV	0.69	0.14
Schmidt-1 (G)	MPMV	0.68	0.11
Schmidt-1 (G)	B-MLV	0.89	0.12
Schmidt-1 (G)	N-MLV	>100	-
Schmidt-1 (G)	EIAV	>100	-
Schmidt-1 (G)	FIV	>100	-
Schmidt-1 (G)	HIV-1	>100	-
Schmidt-1 (G)	HIV-2	>100	-
Schmidt-1 (G)	SIVsmE543	>100	-
Schmidt-1 (G)	SIVmac239	81.96	14.41
Schmidt-1 (G)	SIVstm	31.15	7.44
Schmidt-1 (G)	SIVrcm	>100	-
Schmidt-1 (G)	SIVmus	1.12	0.32
Schmidt-1 (G)	SIVagmVerv	11.46	1.86
Schmidt-1 (G)	SIVagmGrv	3.28	0.52
Schmidt-1 (G)	SIVagmTan	0.84	0.09

DeB (G)	RSV	0.82	0.13
DeB (G)	MPMV	0.71	0.12
DeB (G)	B-MLV	0.88	0.11
DeB (G)	N-MLV	>100	-
DeB (G)	EIAV	>100	-
DeB (G)	FIV	>100	-
DeB (G)	HIV-1	>100	-
DeB (G)	HIV-2	>100	-
DeB (G)	SIVsmE543	>100	-
DeB (G)	SIVmac239	>100	-
DeB (G)	SIVstm	>100	-
DeB (G)	SIVrcm	>100	-
DeB (G)	SIVmus	0.95	0.15

DeB (G)	SIVagmVerv	47.69	7.02
DeB (G)	SIVagmGrv	11.30	2.39
DeB (G)	SIVagmTan	2.58	0.19

Anc-G+20	RSV	1.04	0.21
Anc-G+20	MPMV	1.85	0.17
Anc-G+20	B-MLV	0.96	0.16
Anc-G+20	N-MLV	9.99	0.52
Anc-G+20	EIAV	77.13	8.96
Anc-G+20	FIV	>100	-
Anc-G+20	HIV-1	>100	-
Anc-G+20	HIV-2	40.37	6.20
Anc-G+20	SIVsmE543	19.35	2.81
Anc-G+20	SIVmac239	4.30	0.47
Anc-G+20	SIVstm	2.52	0.08
Anc-G+20	SIVrcm	38.21	9.80
Anc-G+20	SIVmus	2.62	1.18
Anc-G+20	SIVagmVerv	1.11	0.12
Anc-G+20	SIVagmGrv	0.88	0.19
Anc-G+20	SIVagmTan	0.88	0.03

AGM Vero (G+20)	RSV	0.86	0.04
AGM Vero (G+20)	MPMV	0.86	0.11
AGM Vero (G+20)	B-MLV	0.85	0.11
AGM Vero (G+20)	N-MLV	>100	-
AGM Vero (G+20)	EIAV	>100	-
AGM Vero (G+20)	FIV	>100	-
AGM Vero (G+20)	HIV-1	>100	-
AGM Vero (G+20)	HIV-2	>100	-
AGM Vero (G+20)	SIVsmE543	>100	-
AGM Vero (G+20)	SIVmac239	40.50	9.61
AGM Vero (G+20)	SIVstm	51.35	2.33
AGM Vero (G+20)	SIVrcm	81.72	7.47
AGM Vero (G+20)	SIVmus	3.48	0.69
AGM Vero (G+20)	SIVagmVerv	3.16	0.18
AGM Vero (G+20)	SIVagmGrv	1.51	0.60
AGM Vero (G+20)	SIVagmTan	0.89	0.08

AGM Cos (G+20)	RSV	0.62	0.08
AGM Cos (G+20)	MPMV	>100	-
AGM Cos (G+20)	B-MLV	1.20	0.24
AGM Cos (G+20)	N-MLV	>100	-

AGM Cos (G+20)	EIAV	69.84	17.65
AGM Cos (G+20)	FIV	94.10	20.79
AGM Cos (G+20)	HIV-1	>100	-
AGM Cos (G+20)	HIV-2	>100	-
AGM Cos (G+20)	SIVsmE543	>100	-
AGM Cos (G+20)	SIVmac239	>100	-
AGM Cos (G+20)	SIVstm	>100	-
AGM Cos (G+20)	SIVrcm	>100	-
AGM Cos (G+20)	SIVmus	14.07	3.45
AGM Cos (G+20)	SIVagmVerv	10.67	1.01
AGM Cos (G+20)	SIVagmGrv	2.51	0.55
AGM Cos (G+20)	SIVagmTan	1.66	0.15

Human (Q)	RSV	1.67	0.73
Human (Q)	MPMV	1.40	0.13
Human (Q)	B-MLV	1.09	0.03
Human (Q)	N-MLV	>100	-
Human (Q)	EIAV	13.37	0.90
Human (Q)	FIV	4.80	0.40
Human (Q)	HIV-1	1.80	0.26
Human (Q)	HIV-2	10.29	1.74
Human (Q)	SIVsmE543	19.42	2.70
Human (Q)	SIVmac239	0.98	0.16
Human (Q)	SIVstm	1.78	0.13
Human (Q)	SIVrcm	15.58	2.13
Human (Q)	SIVmus	1.85	0.33
Human (Q)	SIVagmVerv	2.32	0.66
Human (Q)	SIVagmGrv	1.30	0.74
Human (Q)	SIVagmTan	0.98	0.04
