# Supplementary material for: Evolutionary and Functional Analysis of Old World Primate TRIM5 Reveals the Ancient Emergence of Primate Lentiviruses and Convergent Evolution Targeting a Conserved Capsid Interface
Source: PLoS Pathog. 2015 Aug 20;11(8):e1005085. doi: 10.1371/journal.ppat.1005085 (PMC4546234; doi:10.1371/journal.ppat.1005085)
Supplement: S2 Dataset — The fold restriction and standard error for each data point in Fig 3. Restriction is abbreviated as “rxn”. Each data point is the average of at least three independent experiments. Values above 100 fold are given as >100, reflecting the limitations of sensitivity of the FACS assay. (PDF) [file ppat.1005085.s011.pdf]

Cell Line	Virus	Average Fold (rxn)	SEM
Anc (Q)	SIVmac239	0.85	0.09
Anc (Q)	HIVnl4.3	64.99	21.41
Anc (Q)	SIVhiv surface	55.45	10.68
Anc (Q)	HIVsiv srurface25	1.01	0.15
Anc (Q)	SIV-V2I	0.99	0.20
Anc (Q)	SIV-Q3V	5.34	1.50
Anc (Q)	SIV-I5N	0.94	0.12
Anc (Q)	SIV-G6L	11.88	0.73
Anc (Q)	SIV-Δ7Q	0.93	0.10
Anc (Q)	SIV-N9Q	0.81	0.10
Anc (Q)	SIV-Y10M	1.17	0.17
Anc (Q)	SIV-Q86V	1.07	0.18
Anc (Q)	SIV-P87H	0.80	0.12
Anc (Q)	SIV-Δ88A	0.89	0.20
Anc (Q)	SIV-A89G	1.50	0.20
Anc (Q)	SIV-Δ91I	1.04	0.18
Anc (Q)	SIV-Q92A	0.97	0.18
Anc (Q)	SIV-Q93P	0.70	0.13
Anc (Q)	SIV-L96M	0.97	0.12
Anc (Q)	SIV-S100R	1.12	0.44
Anc (Q)	SIV-S110T	1.23	0.35
Anc (Q)	SIV-V111L	7.21	1.41
Anc (Q)	SIV-D112Q	1.07	0.20
Anc (Q)	SIV-Q116G	0.71	0.04
Anc (Q)	SIV-Y119T	1.12	0.08
Anc (Q)	SIV-Q121Δ	1.09	0.09
Anc (Q)	SIV-Q122N	0.99	0.28

Rhesus (Q)	SIVmac239	0.76	0.08
Rhesus (Q)	HIVnl4.3	67.40	18.86
Rhesus (Q)	SIVhiv surface	>100	-
Rhesus (Q)	HIVsiv srurface25	0.85	0.21
Rhesus (Q)	SIV-V2I	0.53	0.04
Rhesus (Q)	SIV-Q3V	1.81	0.20
Rhesus (Q)	SIV-I5N	0.55	0.08
Rhesus (Q)	SIV-G6L	4.51	0.72
Rhesus (Q)	SIV-Δ7Q	0.57	0.09
Rhesus (Q)	SIV-N9Q	0.55	0.08
Rhesus (Q)	SIV-Y10M	0.53	0.06
Rhesus (Q)	SIV-Q86V	0.50	0.04
Rhesus (Q)	SIV-P87H	0.55	0.06

Rhesus (Q)	SIV-Δ88A	0.57	0.01
Rhesus (Q)	SIV-A89G	0.56	0.10
Rhesus (Q)	SIV-Δ91I	0.47	0.08
Rhesus (Q)	SIV-Q92A	0.55	0.08
Rhesus (Q)	SIV-Q93P	0.48	0.03
Rhesus (Q)	SIV-L96M	0.51	0.05
Rhesus (Q)	SIV-S100R	0.52	0.04
Rhesus (Q)	SIV-S110T	0.55	0.05
Rhesus (Q)	SIV-V111L	0.52	0.06
Rhesus (Q)	SIV-D112Q	0.57	0.07
Rhesus (Q)	SIV-Q116G	0.51	0.05
Rhesus (Q)	SIV-Y119T	0.57	0.02
Rhesus (Q)	SIV-Q121Δ	0.47	0.02
Rhesus (Q)	SIV-Q122N	0.49	0.03

Anc-SFP	SIVmac239	0.62	0.03
Anc-SFP	HIVnl4.3	92.18	48.54
Anc-SFP	SIVhiv surface	>100	-
Anc-SFP	HIVsiv srurface25	0.62	0.04
Anc-SFP	SIV-V2I	0.63	0.15
Anc-SFP	SIV-Q3V	8.06	2.67
Anc-SFP	SIV-I5N	0.68	0.14
Anc-SFP	SIV-G6L	6.66	0.62
Anc-SFP	SIV-Δ7Q	0.63	0.04
Anc-SFP	SIV-N9Q	0.60	0.07
Anc-SFP	SIV-Y10M	0.64	0.04
Anc-SFP	SIV-Q86V	0.79	0.21
Anc-SFP	SIV-P87H	0.48	0.09
Anc-SFP	SIV-Δ88A	0.62	0.17
Anc-SFP	SIV-A89G	0.72	0.13
Anc-SFP	SIV-Δ91I	0.53	0.06
Anc-SFP	SIV-Q92A	0.60	0.07
Anc-SFP	SIV-Q93P	0.66	0.09
Anc-SFP	SIV-L96M	0.68	0.08
Anc-SFP	SIV-S100R	0.57	0.14
Anc-SFP	SIV-S110T	1.02	0.46
Anc-SFP	SIV-V111L	0.86	0.13
Anc-SFP	SIV-D112Q	0.90	0.24
Anc-SFP	SIV-Q116G	0.67	0.05
Anc-SFP	SIV-Y119T	0.71	0.11
Anc-SFP	SIV-Q121Δ	0.74	0.13
Anc-SFP	SIV-Q122N	0.77	0.08

SM (SFP)	SIVmac239	0.78	0.07
SM (SFP)	HIVnl4.3	9.11	1.43
SM (SFP)	SIVhiv surface	23.78	1.89
SM (SFP)	HIVsiv srurface25	1.01	0.26
SM (SFP)	SIV-V2I	0.73	0.11
SM (SFP)	SIV-Q3V	6.70	1.31
SM (SFP)	SIV-I5N	0.71	0.15
SM (SFP)	SIV-G6L	5.12	1.11
SM (SFP)	SIV-Δ7Q	0.72	0.08
SM (SFP)	SIV-N9Q	0.71	0.10
SM (SFP)	SIV-Y10M	0.69	0.10
SM (SFP)	SIV-Q86V	0.78	0.07
SM (SFP)	SIV-P87H	0.66	0.14
SM (SFP)	SIV-Δ88A	0.66	0.20
SM (SFP)	SIV-A89G	1.09	0.22
SM (SFP)	SIV-Δ91I	0.68	0.16
SM (SFP)	SIV-Q92A	0.79	0.14
SM (SFP)	SIV-Q93P	0.82	0.28
SM (SFP)	SIV-L96M	0.98	0.20
SM (SFP)	SIV-S100R	0.96	0.42
SM (SFP)	SIV-S110T	1.04	0.25
SM (SFP)	SIV-V111L	0.95	0.12
SM (SFP)	SIV-D112Q	0.92	0.11
SM (SFP)	SIV-Q116G	0.77	0.05
SM (SFP)	SIV-Y119T	0.89	0.17
SM (SFP)	SIV-Q121Δ	0.83	0.05
SM (SFP)	SIV-Q122N	0.78	0.15

Anc-TFP	SIVmac239	0.71	0.03
Anc-TFP	HIVnl4.3	>100	-
Anc-TFP	SIVhiv surface	>100	-
Anc-TFP	HIVsiv srurface25	2.27	1.18
Anc-TFP	SIV-V2I	0.80	0.14
Anc-TFP	SIV-Q3V	11.00	3.94
Anc-TFP	SIV-I5N	0.94	0.10
Anc-TFP	SIV-G6L	11.89	2.02
Anc-TFP	SIV-Δ7Q	1.59	0.31
Anc-TFP	SIV-N9Q	0.80	0.20
Anc-TFP	SIV-Y10M	1.01	0.24
Anc-TFP	SIV-Q86V	1.04	0.17
Anc-TFP	SIV-P87H	0.72	0.06

Anc-TFP	SIV-Δ88A	0.93	0.21
Anc-TFP	SIV-A89G	1.42	0.20
Anc-TFP	SIV-Δ91I	1.05	0.29
Anc-TFP	SIV-Q92A	0.70	0.02
Anc-TFP	SIV-Q93P	0.88	0.18
Anc-TFP	SIV-L96M	1.34	0.37
Anc-TFP	SIV-S100R	1.08	0.28
Anc-TFP	SIV-S110T	0.94	0.36
Anc-TFP	SIV-V111L	1.82	0.30
Anc-TFP	SIV-D112Q	1.67	0.39
Anc-TFP	SIV-Q116G	0.78	0.03
Anc-TFP	SIV-Y119T	1.08	0.17
Anc-TFP	SIV-Q121Δ	1.09	0.22
Anc-TFP	SIV-Q122N	0.86	0.13

Rhesus (TFP)	SIVmac239	1.13	0.06
Rhesus (TFP)	HIVnl4.3	>100	-
Rhesus (TFP)	SIVhiv surface	>100	-
Rhesus (TFP)	HIVsiv srurface25	1.92	0.25
Rhesus (TFP)	SIV-V2I	1.35	0.16
Rhesus (TFP)	SIV-Q3V	8.51	0.41
Rhesus (TFP)	SIV-I5N	0.74	0.09
Rhesus (TFP)	SIV-G6L	15.33	0.89
Rhesus (TFP)	SIV-Δ7Q	10.42	1.31
Rhesus (TFP)	SIV-N9Q	1.70	0.33
Rhesus (TFP)	SIV-Y10M	3.59	0.43
Rhesus (TFP)	SIV-Q86V	3.28	0.34
Rhesus (TFP)	SIV-P87H	6.60	0.56
Rhesus (TFP)	SIV-Δ88A	1.10	0.03
Rhesus (TFP)	SIV-A89G	4.00	0.55
Rhesus (TFP)	SIV-Δ91I	4.51	0.86
Rhesus (TFP)	SIV-Q92A	0.89	0.11
Rhesus (TFP)	SIV-Q93P	2.60	0.15
Rhesus (TFP)	SIV-L96M	7.04	1.06
Rhesus (TFP)	SIV-S100R	20.71	3.45
Rhesus (TFP)	SIV-S110T	1.39	0.11
Rhesus (TFP)	SIV-V111L	18.74	3.87
Rhesus (TFP)	SIV-D112Q	7.66	0.41
Rhesus (TFP)	SIV-Q116G	2.53	0.21
Rhesus (TFP)	SIV-Y119T	1.05	0.03
Rhesus (TFP)	SIV-Q121Δ	2.03	0.06
Rhesus (TFP)	SIV-Q122N	0.81	0.07

Anc-G	SIVmac239	0.67	0.06
Anc-G	HIVnl4.3	>100	-
Anc-G	SIVhiv surface	>100	-
Anc-G	HIVsiv srurface25	5.70	0.99
Anc-G	SIV-V2I	0.68	0.18
Anc-G	SIV-Q3V	9.45	4.31
Anc-G	SIV-I5N	1.02	0.27
Anc-G	SIV-G6L	14.68	2.71
Anc-G	SIV-Δ7Q	1.57	0.22
Anc-G	SIV-N9Q	0.73	0.11
Anc-G	SIV-Y10M	0.94	0.20
Anc-G	SIV-Q86V	1.09	0.18
Anc-G	SIV-P87H	1.15	0.29
Anc-G	SIV-Δ88A	0.60	0.08
Anc-G	SIV-A89G	1.91	0.34
Anc-G	SIV-Δ91I	1.72	0.75
Anc-G	SIV-Q92A	1.87	1.04
Anc-G	SIV-Q93P	1.56	0.84
Anc-G	SIV-L96M	1.00	0.31
Anc-G	SIV-S100R	1.23	0.48
Anc-G	SIV-S110T	1.11	0.34
Anc-G	SIV-V111L	6.02	1.69
Anc-G	SIV-D112Q	2.43	0.41
Anc-G	SIV-Q116G	0.73	0.14
Anc-G	SIV-Y119T	0.89	0.05
Anc-G	SIV-Q121Δ	1.06	0.29
Anc-G	SIV-Q122N	0.84	0.17

Mus (G)	SIVmac239	1.47	0.03
Mus (G)	HIVnl4.3	>100	-
Mus (G)	SIVhiv surface	>100	-
Mus (G)	HIVsiv srurface25	9.07	2.32
Mus (G)	SIV-V2I	1.05	0.29
Mus (G)	SIV-Q3V	5.26	2.20
Mus (G)	SIV-I5N	1.23	0.21
Mus (G)	SIV-G6L	12.45	5.23
Mus (G)	SIV-Δ7Q	3.25	0.24
Mus (G)	SIV-N9Q	1.10	0.18
Mus (G)	SIV-Y10M	1.59	0.38
Mus (G)	SIV-Q86V	2.33	0.54
Mus (G)	SIV-P87H	13.12	1.13

Mus (G)	SIV-Δ88A	0.85	0.20
Mus (G)	SIV-A89G	4.00	0.91
Mus (G)	SIV-Δ91I	3.99	0.84
Mus (G)	SIV-Q92A	1.01	0.05
Mus (G)	SIV-Q93P	2.54	0.61
Mus (G)	SIV-L96M	2.40	0.56
Mus (G)	SIV-S100R	3.43	1.12
Mus (G)	SIV-S110T	1.11	0.20
Mus (G)	SIV-V111L	6.24	1.07
Mus (G)	SIV-D112Q	5.96	0.86
Mus (G)	SIV-Q116G	1.72	0.40
Mus (G)	SIV-Y119T	1.04	0.14
Mus (G)	SIV-Q121Δ	1.57	0.27
Mus (G)	SIV-Q122N	0.88	0.19

Schmidt-2 (G)	SIVmac239	2.83	0.10
Schmidt-2 (G)	HIVnl4.3	>100	-
Schmidt-2 (G)	SIVhiv surface	>100	-
Schmidt-2 (G)	HIVsiv srurface25	10.22	2.08
Schmidt-2 (G)	SIV-V2I	1.62	0.13
Schmidt-2 (G)	SIV-Q3V	4.64	1.40
Schmidt-2 (G)	SIV-I5N	1.35	0.30
Schmidt-2 (G)	SIV-G6L	17.96	6.15
Schmidt-2 (G)	SIV-Δ7Q	8.74	3.65
Schmidt-2 (G)	SIV-N9Q	1.72	0.62
Schmidt-2 (G)	SIV-Y10M	2.63	0.15
Schmidt-2 (G)	SIV-Q86V	4.04	0.79
Schmidt-2 (G)	SIV-P87H	21.81	5.79
Schmidt-2 (G)	SIV-Δ88A	1.14	0.12
Schmidt-2 (G)	SIV-A89G	12.27	1.73
Schmidt-2 (G)	SIV-Δ91I	8.57	2.23
Schmidt-2 (G)	SIV-Q92A	1.30	0.25
Schmidt-2 (G)	SIV-Q93P	5.92	1.56
Schmidt-2 (G)	SIV-L96M	4.76	1.42
Schmidt-2 (G)	SIV-S100R	5.92	1.61
Schmidt-2 (G)	SIV-S110T	1.73	0.58
Schmidt-2 (G)	SIV-V111L	11.21	1.56
Schmidt-2 (G)	SIV-D112Q	9.70	1.47
Schmidt-2 (G)	SIV-Q116G	3.74	0.59
Schmidt-2 (G)	SIV-Y119T	1.13	0.34
Schmidt-2 (G)	SIV-Q121Δ	2.65	0.30
Schmidt-2 (G)	SIV-Q122N	1.14	0.24

Wolf's (G)	SIVmac239	0.84	0.03
Wolf's (G)	HIVnl4.3	1.73	0.27
Wolf's (G)	SIVhiv surface	1.45	0.17
Wolf's (G)	HIVsiv srurface25	0.68	0.19
Wolf's (G)	SIV-V2I	0.45	0.05
Wolf's (G)	SIV-Q3V	0.64	0.02
Wolf's (G)	SIV-I5N	0.37	0.02
Wolf's (G)	SIV-G6L	0.75	0.04
Wolf's (G)	SIV-Δ7Q	0.50	0.02
Wolf's (G)	SIV-N9Q	0.48	0.02
Wolf's (G)	SIV-Y10M	0.45	0.01
Wolf's (G)	SIV-Q86V	0.52	0.07
Wolf's (G)	SIV-P87H	0.43	0.03
Wolf's (G)	SIV-Δ88A	0.43	0.07
Wolf's (G)	SIV-A89G	0.46	0.04
Wolf's (G)	SIV-Δ91I	0.40	0.05
Wolf's (G)	SIV-Q92A	0.47	0.02
Wolf's (G)	SIV-Q93P	0.41	0.02
Wolf's (G)	SIV-L96M	0.46	0.04
Wolf's (G)	SIV-S100R	0.45	0.04
Wolf's (G)	SIV-S110T	0.48	0.04
Wolf's (G)	SIV-V111L	0.53	0.09
Wolf's (G)	SIV-D112Q	0.50	0.04
Wolf's (G)	SIV-Q116G	0.44	0.02
Wolf's (G)	SIV-Y119T	0.54	0.06
Wolf's (G)	SIV-Q121Δ	0.48	0.04
Wolf's (G)	SIV-Q122N	0.46	0.04
